# Global grassland productivity and carbon storage benefit from functional diversity under climate change

**DOI:** 10.1073/pnas.2528067123

**Published:** 2026-10-07

**Authors:** Stephen Björn Wirth, Christoph Müller, Friedhelm Taube, Jens Heinke, Britta Tietjen, Susanne Rolinski

**Affiliations:** ^a^ Potsdam Institute for Climate Impact Research, Member of the Leibniz Association, Potsdam 14412, Germany; ^b^ Institute of Crop Science and Plant Breeding, Grass and Forage Science/Organic Agriculture, Faculty of Agricultural and Nutritional Sciences, Kiel University, Kiel 24118, Germany; ^c^ Theoretical Ecology, Institute of Biology, Department of Biology, Chemistry, and Pharmacy, Freie Universität Berlin, Berlin 14195, Germany; ^d^Brandenburg Institute of Advanced Biodiversity Research, Berlin D-14195, Germany

**Keywords:** managed grasslands, soil organic carbon, functional diversity, dynamic global vegetation model

## Abstract

Functional diversity plays an important role for ecosystem services of managed grasslands such as their gross primary productivity (GPP) and soil organic carbon (SOC) stocks. Yet vegetation models assessing functional diversity have been limited to small spatial scales. We used the Lund-Potsdam-Jena managed land (LPJmL) competitor stress-tolerant ruderal (CSR) model to assess the role of functional diversity for managed grassland GPP and SOC under climate change at the global scale. LPJmL-CSR simulates functional diversity using plant functional types representing different ecological strategies based on functional traits connected to CSR strategies. Our simulation experiments assumed low and high functional diversity levels and moderate and strong climate change scenarios. Results show that functional diversity plays an important role for GPP and SOC stocks already under moderate climate change. Strong loss of functional diversity prohibits communities’ adaptation to climate change, resulting in loss of GPP and SOC. Spatial patterns of GPP and SOC stocks show substantial differences between the low and high functional diversity scenarios dependent on regional conditions. These results are consistent with empirical findings and support the importance of functional diversity as insurance against climate change. The results of our global-scale model assessment show that enhancing functional diversity could be crucial for maintaining grassland productivity and thus human well-being under global change. In combination with empirical findings, they underpin the need for management practices that support functional diversity of managed grasslands.

## Significance

The ecosystem services of managed grasslands and rangelands depend strongly on the diversity of plant traits within these ecosystems. This “functional diversity” influences their productivity as well as resistance and resilience against climate change. Yet, global vegetation models commonly only distinguish C3 and C4 plant functional types and do not explicitly consider the role of functional diversity in managed grasslands and rangelands. We provide a global-scale assessment that shows the importance of functional diversity to maintain productivity and soil organic carbon stocks of managed grassland and rangelands under climate change. Despite our simplified functional diversity approach, this highlights the need to account for functional diversity when conducting climate impact research as well as designing respective policies.

Approximately 25% of the terrestrial ice-free Earth surface is covered by permanent grassland or rangeland ecosystems including a variety of grassland systems ranging from lush humid meadows to dry steppe systems (1). These ecosystems provide important ecosystem services supporting the livelihoods of at least a billion people (2). However, as a consequence of intensive land use, and land abandonment, grassland ecosystems are at risk of degradation, a risk that will likely increase under climate change (3, 4). Additionally, climate change may increase grassland ecosystems’ vulnerability to overgrazing and ecosystem services may decline (5). To prevent further grassland degradation, management adaptations need to be developed based on improved understanding of the processes underpinning ecosystem service provisioning. Climatic conditions, soil health, and management are all important factors for grassland ecosystem services. Temperature, soil water, and nutrient availability directly control the amount of carbon grassland ecosystems can assimilate. While management in the form of irrigation or fertilizer application can be used to increase carbon assimilation by reducing water or nutrient stress, grazing, or mowing reduces above-ground biomass (6, 7). However, the effect of grazing or mowing on carbon assimilation is complex. Low to intermediate grazing or mowing intensities can have a positive effect on above-ground biomass, for example through the reduction of inter- and intraspecific competition for space and light. However, higher intensities lead to a decline of above-ground biomass and ecosystem carbon assimilation in the long-term (e.g., refs. 8 and 9) assuming the removed biomass is used as feed for livestock. In addition to climatic, soil, and management conditions, the functional diversity of a grassland ecosystem also contributes to ecosystem service provision in rainfed and unfertilized grasslands. This is especially important under environmental stress such as drought conditions. As a consequence, functional diversity has been discussed as a means to increase resilience and/or resistance against climate change impacts (10, 11).

The role of functional diversity has been extensively researched at small spatial scales using field experiments and observations (e.g., refs. 12–14) and synthesized in meta-analyses (e.g., refs. 15 and 16). At the local to landscape scales the role of functional diversity has also been incorporated into simulation models. Individual-based models such as GRASSMIND (e.g., refs. 17 and 18), IBC-grass (e.g., refs. 19 and 20) or aDGVM2 (e.g., refs. 21 and 22) simulate functional diversity via competition between individual plants for which key functional traits are sampled from a distribution. Such models have successfully been applied to simulate the importance of functional diversity for several ecosystem services under different climatic and management conditions. These studies provide important insight and advance research on universal relationships explaining observed benefits of functional diversity (23). However, large- or global-scale studies that quantify the role of functional diversity for grassland ecosystem services have not been conducted. For such large scale analyses, dynamic global vegetation models are widely applied. However, functional diversity of managed grasslands is not represented in state-of-the-art dynamic global vegetation models despite the extent of grassland areas.

Dynamic global vegetation models are widely applied to study the effect of climate change on vegetation dynamics and the carbon cycle (e.g., refs. 24 and 25). Historically, dynamic global vegetation models have represented vegetation using the concept of plant functional types (PFTs) neglecting functional diversity. While tree PFTs are at least distinguished via different leaf types and phenology, grasslands are usually simulated using a single PFT for a specific climate. For example, the models evaluated by Wilcox et al. (26) and LPJmL only distinguish C3 and C4 PFTs with barely or not overlapping growing areas. However, this implies the assumption that only environmental conditions and management control grassland productivity, neglecting the role of functional diversity.

Recently, the LPJmL model (27–30) has been extended to simulate grassland functional diversity based on CSR theory (31) (*SI Appendix*, *Overview of LPJmL-CSR*). This extension contributes to some of the key areas for model improvement (e.g., reproduction, competition for and uptake of water and nutrients, and spatial dynamics) identified by Wilcox et al. (26). The CSR theory distinguishes three main ecological strategies: the competitive (C) strategy is associated with efficient resource use, fast growth, and low tolerance toward stress. The stress-tolerant (S) strategy is associated with investment into more expensive but robust tissue, slow growth, and high stress tolerance. The ruderal (R) strategy is associated with a rapid growth in periods of favorable conditions between disturbances. During these periods, it completes its life cycle. As a consequence, it has a high disturbance tolerance but no stress tolerance. Intermediate strategies that show characteristics of several of the main strategies also exist. While the CSR theory by itself is not a representation of functional diversity, it has been connected to the global spectrum of plant form and function through functional traits (32). Representing these functional traits as model parameters enables the LPJmL-CSR model to simulate key trade-offs present in the global spectrum of plant form and function (33). The model reduces this complexity to the differences between strategies described by CSR theory. LPJmL-CSR was extended by a C-, S-, and R-PFT to represent the three main strategies of the CSR theory (34). Intermediate strategies are not explicitly considered and are only reflected in the resulting mixture of the C-, S-, and R-PFTs. LPJmL-CSR enables climate change impact assessments that explicitly consider grassland functional diversity to quantify its role for future grassland ecosystem services.

We limit our scope to two highly studied ecosystem services of grasslands, the GPP, which is important for forage supply of grazing animals, and the SOC stock, which contributes to climate change mitigation. Environmental conditions and management are the key drivers of both productivity and SOC storage, but functional diversity is crucial as well (e.g., refs. 12 and 35). Important factors controlling productivity and SOC dynamics are radiation, temperature, soil water and nutrient status, and the atmospheric ${\mathrm{CO}}_{2}$ concentration. All those factors will likely be altered by climate change. For example, a reduction of the ocean carbon sink as well as an increased risk of wildfires will likely contribute to increasing atmospheric ${\mathrm{CO}}_{2}$ concentrations in addition to anthropogenic emissions (36, 37). The global average radiative forcing is projected to increase, which will increase, temperature, and annual precipitation over land (5). However, spatial and temporal patterns will also change and precipitation will likely decrease over large parts of the subtropics while increasing over higher latitudes (5). These changes will also affect competitive and facilitative processes in managed grassland communities.

Several of the climatic variables affected by climate change control managed grassland productivity. Grazing or mowing removes biomass, which reduces competition for light. Light is therefore not strongly limiting productivity. Rising atmospheric ${\mathrm{CO}}_{2}$ concentrations will potentially lead to increasing productivity and SOC stocks (38). Generally, high grassland productivity can be achieved at moderate temperatures and sufficient water and nutrient supply but may be severely limited by drought and nutrient stress. High productivity leads to high biomass input into the soil through litterfall. This can lead to a high SOC storage, especially since the formation of SOC-stabilizing mineral surfaces and decomposition rates are also affected by precipitation. However, this depends on the temperature. High temperatures lead to increased microbial activity and therefore decomposition. These mechanisms and their role for grassland productivity and SOC stocks have already been modeled at large scales (e.g., refs. 39 and 40). However, we believe that explicitly including the role of functional diversity in such assessments will improve projections of managed grassland productivity and SOC stocks under climate change.

Using the LPJmL-CSR model, we conducted a global assessment of the role of functional diversity for grassland productivity and SOC storage in grasslands. In our assessment, we compare a world with high and low functional diversity against each other to quantify the role of functional diversity. We focus our assessment on trends and spatial patterns of productivity and SOC stocks globally and in exemplary regions that showcase prominent differences to answer the following research questions:

- To what extent does functional diversity contribute to the world’s grassland productivity and SOC stocks under climate change?
- How does the presence or absence of functional diversity shape the main spatial patterns and regional differences of grassland productivity and SOC stocks?
- How do the model results compare to empirical findings on the role of functional diversity in different regions of the world?

To address our research questions, we used the global vegetation model LPJmL-CSR under different functional diversity settings. We simulated two different functional diversity scenarios using climate projections from five general circulation models for two forcing scenarios. Scenarios names follow the conventions described in ref. 41 and combine a shared socioeconomic pathway (SSP) and a radiative forcing level.

We use two scenarios: SSP 1-2.6 is a low-emission scenario with an average global temperature increase below 2^°^C. SSP 3-7.0 is a high-emission scenario where global average temperature increase is around 4 ^°^C. For LPJmL-CSR socioeconomic forcing variables are not considered. Consequently, there are substantial differences in the following variables used here to drive the simulations: daily average, minimum and maximum temperature, precipitation, radiative forcing, windspeed, and atmospheric CO_2_ concentration. For both forcing scenarios we simulated two functional diversity scenarios:.

- **FD**−: where we remove all nondominant PFTs in each grid cell at the end of the historical period and prohibit establishment of new PFTs.
- **FD**+: where we maintain the PFTs established (nonzero foliage projective cover) at the end of the historical period but also allow establishment of new PFTs.

## Results

### Global Productivity and SOC Stocks Under Climate Change.

**Fig. 1.**
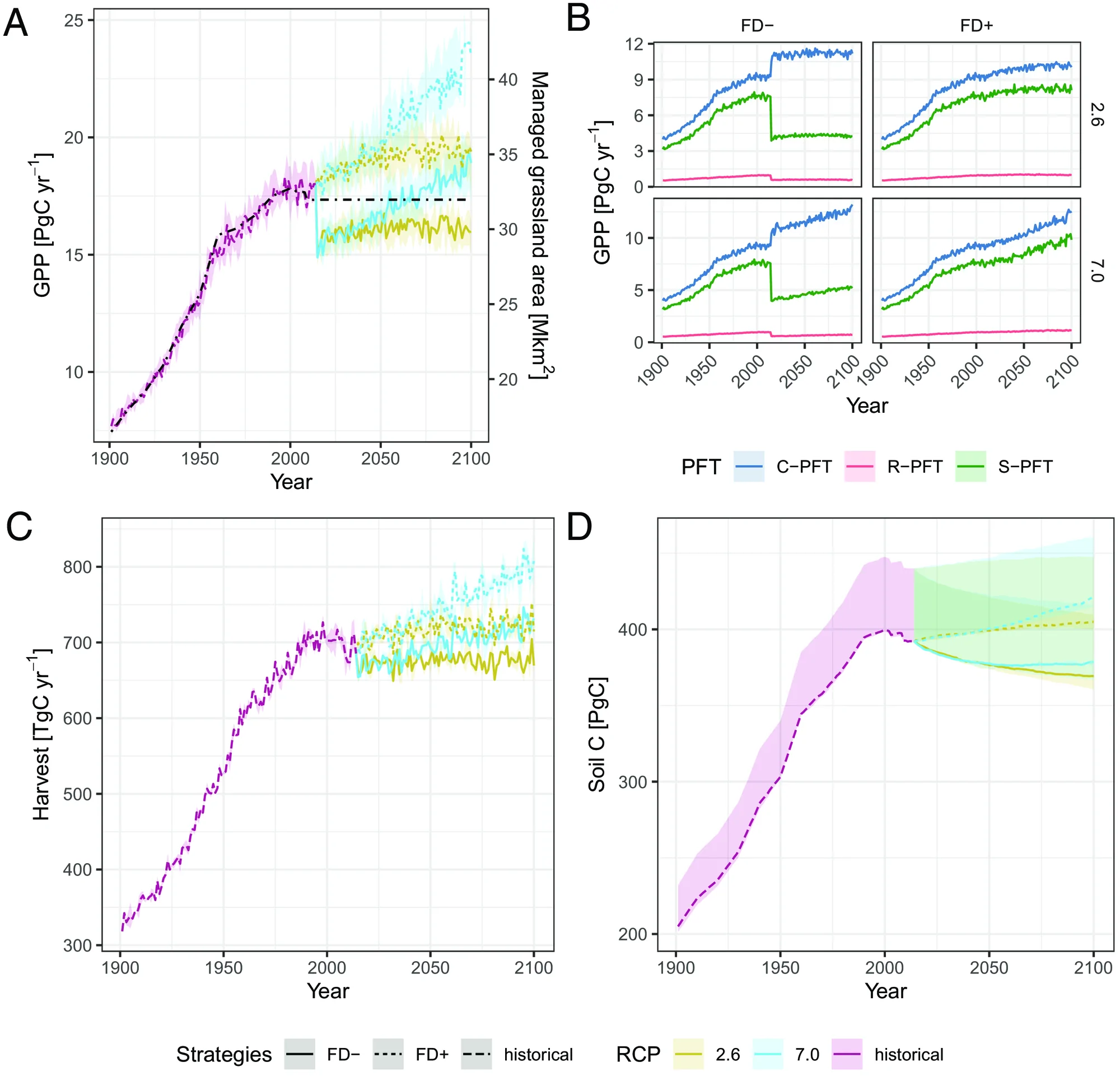
Timeseries of global total GPP (*A*), PFT-specific GPP (*B*), dry matter intake (*C*), and SOC stocks (*D*) from 1901 until 2100. Panels *A*, *C*, and *D* show historical data (purple, dashed) and projections for SSP 1-2.6 (yellow) and SSP 3-7.0 (lightblue) for the FD+ (solid) and FD− (dotted) scenarios. Grassland areas are shown in panel *A* (dot-dashed). For the PFT-specific GPP, different scenarios (FD− and FD+) and SSPs (1-2.6 and 3-7.0) are shown in separate panels and colors distinguish the C- (darkblue), S- (red), and R-PFT (darkgreen). Lines show the median and shaded areas the 5th to 95th percentile across five general circulation models.

For the historical period, LPJmL-CSR simulates a strong increase of grassland GPP (Fig. 1*A*). This increase is predominantly caused by increasing grassland areas. Increasing atmospheric $CO_{2}$ concentration and N inputs from deposition also contribute to some extent. Total global grassland GPP and dry matter intake were substantially higher in FD+ compared to FD− for both SSP 1-2.6 and SSP 3-7.0 (Fig. 1 *A* and *C*). GPP was approximately 20 $\mathrm{PgC}\;y^{-1}$ in 2100 for SSP 1-2.6 and 25 $\mathrm{PgC}\;y^{-1}$ for SSP 3-7.0 for the FD+ scenario (Fig. 1 a). Independent of the functional diversity scenario, LPJmL-CSR simulated a stronger GPP increase under SSP 3-7.0 compared to SSP 1-2.6. This results from multiple factors: CO_2_ fertilization, adaptation of community composition favoring PFTs with higher temperature optima for photosynthesis (*SI Appendix*, *Photosynthesis Module*) and potential underestimation of drought mortality (*Discussion* and *SI Appendix*, *Overview of LPJmL-CSR*). This calls for additional analysis and future model development. However, we here focus on the differences between the scenario with functional diversity (FD+) and without functional diversity (FD−).

For FD− GPP was substantially lower compared to FD+, which reproduces the well-established link between functional diversity and productivity. GPP reductions are reflected in lower dry matter intake and a decline in SOC (Fig. 1 *C* and *D*). PFT-specific GPP shows that the lower overall GPP in the FD− scenarios resulted from a stronger GPP decrease of the S- and R-PFT that outweighed the GPP increase of the C-PFT (Fig. 1*B*). Essentially, the S- and R-PFTs were more often subordinate or marginal and therefore removed in the FD− scenario. However, while this made additional resources available for the dominant C-PFT, leading to an increase, this cannot compensate the losses from removing the S- and R-PFTs.

**Fig. 2.**
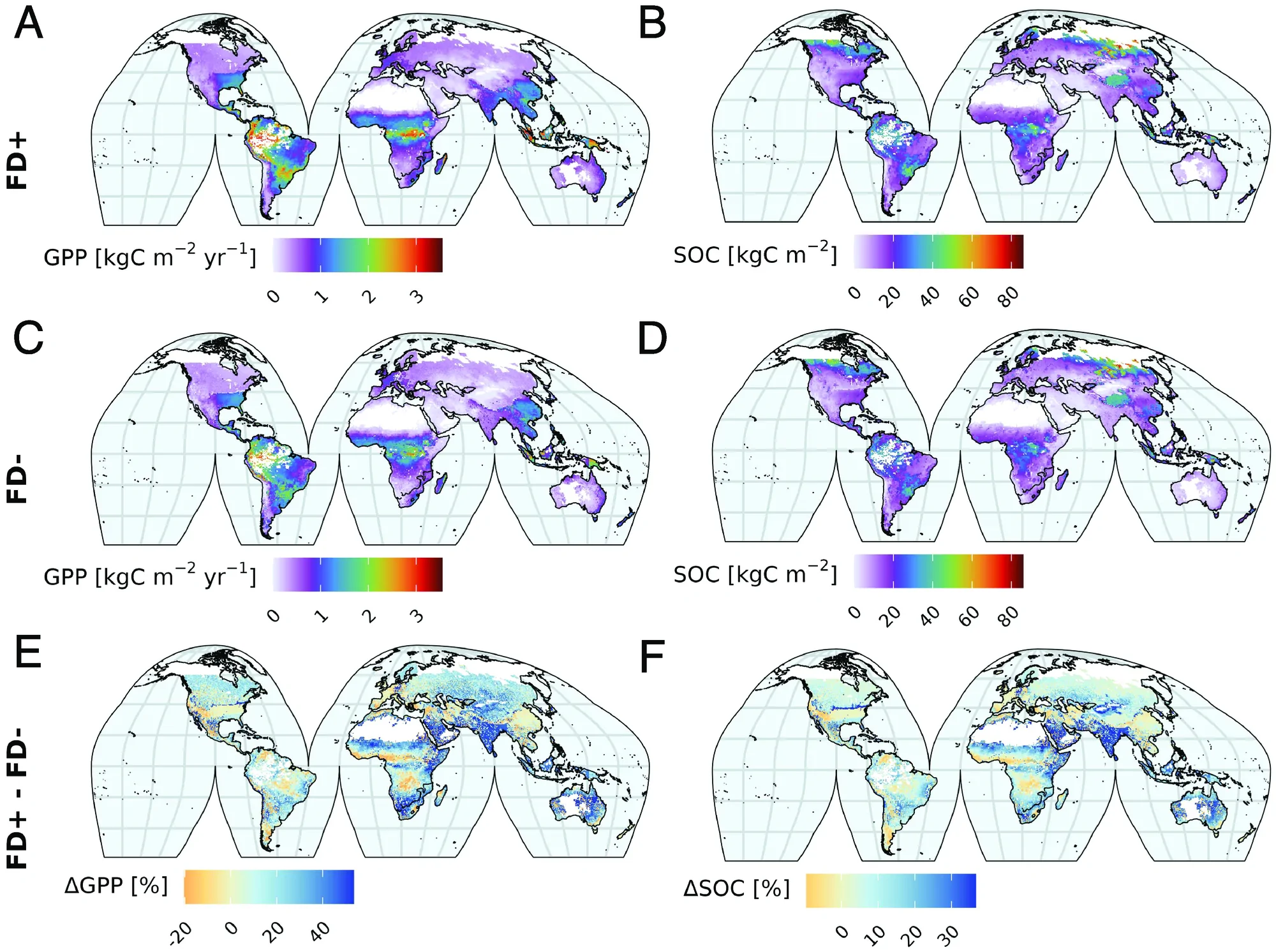
Global distribution of GPP and SOC dependent on functional diversity. *Left* panels show GPP and right panels show SOC. Panels show the average GPP and SOC for FD+ (*A,B*), FD− (*C,D*), and their difference (*E,F*), i.e., the net effect of higher functional diversity. All distributions show the 2091 to 2100 average of the respective output variable simulated using climate data from the MRI-ESM2-0.

The spatial patterns of SSP 1-2.6 and SSP 3-7.0 are similar (compare Fig. 2 and *SI Appendix*, Fig. S6). Results mainly differ in two ways: the extent of the increases in GPP, SOC, and other variables over time and the relative difference between FD+ and FD−. (Fig. 1 and *SI Appendix*, Figs. S6 and S7). Fig. 1 shows that SSP 3-7.0 results in stronger increases in both GPP and SOC compared to SSP 1-2.6 for both FD+ and FD− by the end of the century (2091 to 2100). However, the spatial patterns differ substantially by region (*SI Appendix*, Fig. S8). GPP and SOC are generally higher not only in FD+ but also in SSP 3-7.0. Fig. S8 shows the differences between SSP 3-7.0 and SSP 1-2.6 at the end of the century for FD+ and FD− separately. Spatial patterns of GPP and SOC are similar and show positive or negligible differences. The strongest differences between SSP 3-7.0 and SSP 1-2.6 occur in the tropics (e.g., the Congo Basin and the Amazon), where temperature increases are highest. In contrast, GPP differences are smaller or absent in water-limited regions (e.g., the Sahel zone and central Australia). This spatial pattern shows that in LPJmL, temperature and $CO_{2}$ fertilization drive productivity gains where water is not limiting, while drought constraints prevent benefits in arid regions.

While the similarity in community response is limited by our coarse representation of functional diversity (*Discussion*), the benefit of functional diversity (FD+ vs. FD−) remains similar between SSP 1-2.6 and SSP 3-7.0. This indicates that functional diversity consistently supports GPP and SOC in both emissions scenarios and allows us to focus on one of the two forcing scenarios in the main text. Therefore, we only show the results for SSP 1-2.6 from here on. Figures for SSP 3-7.0 are provided in the SI. All maps show data for MRI-ESM2-0 only because the spatial patterns of an average or the median across general circulation models are not meaningful. Generally, spatial patterns did not differ strongly between general circulation models and showing only one example is sufficient to support our findings.

### Spatial Patterns of Productivity and SOC Stocks at the End of the 21st Century.

Spatial patterns of GPP are similar between FD+ and FD− with highest GPP in the inner tropics and lowest GPP in semiarid, arid, and polar regions (Fig. 2 *A* and *C*). The strongest GPP reduction occurred around the equator, Paraguay and India where GPP is up to 50% lower for the FD− scenario (Fig. 2*E*). GPP increased up to 20% in FD− in the African countries north and south of the equator and in Spain.

SOC patterns are also similar between FD+ and FD− with highest values in polar regions where decay rates are low and lower values in drier and/or warmer climates (Fig. 2 *B* and *D*). Following the reduction of GPP, highest SOC differences between FD+ and FD− were simulated around the equator, in Paraguay and in India (Fig. 2). Similarly, SOC slightly increased in areas where GPP increased.

To assess how the role of functional diversity differs between climate scenarios, we compare the spatial patterns of FD+ minus FD− for the 2091 to 2100 average under both SSP 1-2.6 and SSP 3-7.0 (Fig. 2 *E* and *F* vs. *SI Appendix*, Fig. S6 *E* and *F*). Comparing Fig. 2 *E* and *F* for SSP 1-2.6 and *SI Appendix*, Fig. S6 *E* and *F* for SSP 3-7.0 shows similar patterns in most areas. The difference between FD+ and FD− is consistent across scenarios. However, some differences emerge in areas where temperature increases limit suitability for polar PFTs. This is most prominently visible in Canada, where GPP and SOC differences between FD+ and FD− are substantially larger in SSP 1-2.6 compared to SSP 3-7.0. The tropical–temperate border regions show a similar but less pronounced pattern for example in Morocco and northern Algeria.

**Fig. 3.**
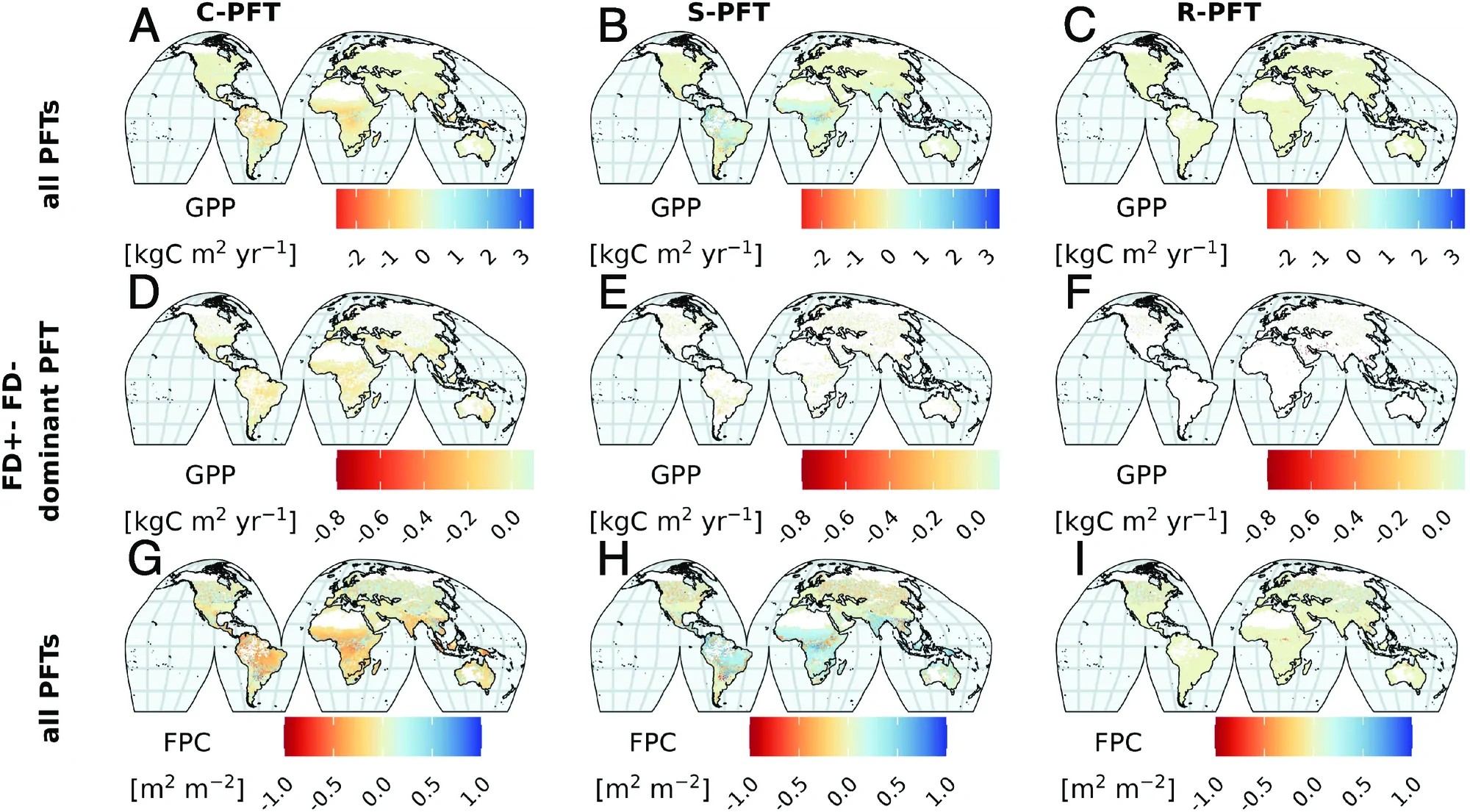
Global distribution of PFT-specific GPP and foliage projective cover dependent on functional diversity. All panels show the absolute change between FD+ and FD−, i.e., the net effect of a higher functional diversity. Row one shows the distribution using all PFTs present in FD+ for the C− (*A*), S− (*B*), and R-PFTs (*C*). Row two shows the distribution using only the dominant PFT in FD+ for the C− (*D*), S− (*E*), and R-PFTs (*F*), where white areas indicate grid cells in which the respective PFT is not dominant and no value is available. Row three shows the distribution using all PFTs present in FD+ for the C− (*G*), S− (*H*), and R-PFTs (I). Similar values (difference of 0) are shown in green. All distribution shows the 2091 to 2100 average of the respective output variable simulated using climate data from the MRI-ESM2-0.

**Fig. 4.**
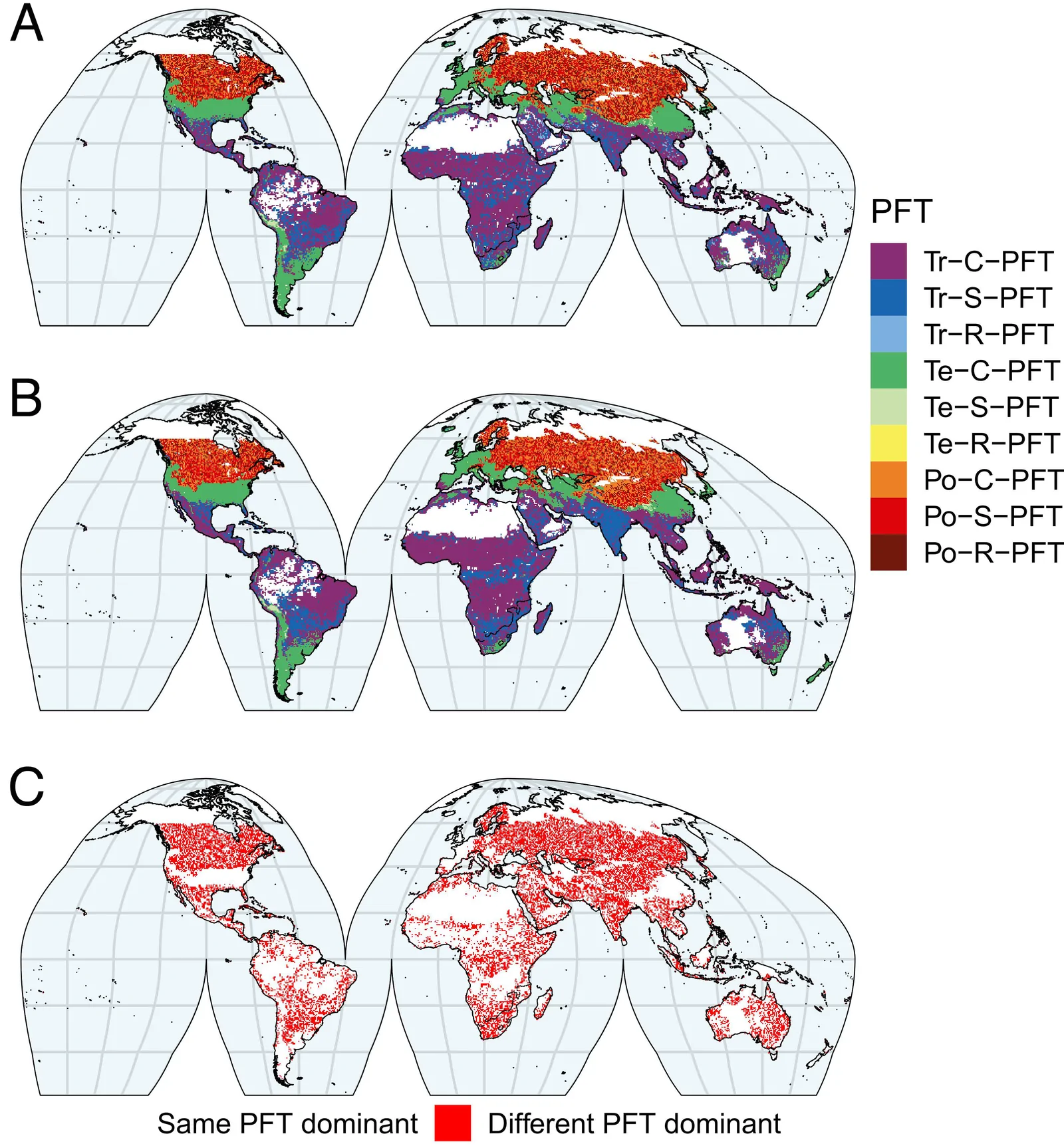
Dominant PFT in FD+ at the end of the historical time period (2014, *A*), the current century (2091 to 2100 average, *B*) and change of the dominant PFT between the end of the historical time period and the current century (*C*). PFTs are the tropical (Tr), temperate (Te) and polar (Po), competitive (C), stress-tolerant (S), and ruderal (R).

Comparing the GPP of FD+ and FD− for the C-, S-, and R-PFTs shows contrasting patterns for the C- and S-PFTs. In large areas, different PFTs are dominant at the end of the century compared to the end of the historical time period across all climates (Fig. 4). For the C-PFTs, LPJmL-CSR simulated higher GPP in FD− compared to FD+ in parts of the tropics, subtropics, the south-east USA, Portugal, Spain, France, and north-west Germany (up to approximately 2 $\mathrm{kgC}\;m^{-2}\;y^{-1}$). In contrast GPP was lower at lower latitudes (up to approximately 2.5 $\mathrm{kgC}\;m^{-2}\;y^{-1}$) (Fig. 3*A*). At the level of C-, S-, and R-PFTs, the losses are likely caused by the removal of the respective PFT and do not have a high informational value. However, increases do not reflect the addition of a PFT but an increase in the productivity of the dominant PFT. In the tropics and subtropics, the C-PFT is dominant. However, the S- and/or R-PFT also make up a substantial share of the community (Fig. S9 and 10 a-c). Removing the subordinate PFTs frees up resources, which can be utilized by the dominant C-PFTs. This is not the case in areas where the C-PFTs were strongly dominant anyway such as the south-east of the United States, Uruguay, southern Brazil, and east China (Fig. 3*G*).

For the S-PFTs, GPP values are up to approximately 3.5 $\mathrm{kgC}\;m^{-2}\;y^{-1}$ lower in the tropics and subtropics and up to 2.5 $\mathrm{kgC}\;m^{-2}\;y^{-1}$ higher in higher latitudes (Fig. 3*B*). The losses are mainly a reflection of areas where the C-PFT was dominant but the S-PFT also made up a substantial share of the community resulting in a large loss of GPP when the S-PFTs were removed (Fig. 3*H*). As a consequence of the removal, the C-PFT can utilize additional resources and increase its GPP if the subordinate S-PFT is removed. However, as described above and shown in Fig. 2, the total GPP is lower in a majority of the subtropics and tropics in FD−.

For the R-PFTs, the differences are generally smaller and limited to latitudes above 40^°^N and west China (Fig. 3*C*). However, the R-PFT is subordinate to the C- or S-PFT in the majority of grid cells (Fig. 3*I*) resulting in a GPP loss of up to approximately 0.5 $\mathrm{kgC}\;m^{-2}\;y^{-1}$. In the few grid cells in which the R-PFT is dominant, the GPP increases by up to approximately 1 $\mathrm{kgC}\;m^{-2}\;y^{-1}$ in FD− compared to FD+.

We assessed whether GPP losses in FD− resulted solely by the removal of subordinate and marginal PFTs. Alternatively, losses could result from reduced productivity of the dominant PFT itself. To test this we compared the GPP of the dominant PFT (selected for FD−) between FD− an FD+ (Fig. 3*D*–*F*). For all strategies, the GPP of the dominant PFT was higher in FD− than in FD+ across large areas. This means that the dominant PFT was less productive when competing with other PFTs in FD+. For the C-PFTs, GPP is up to approximately 0.2 $\mathrm{kgC}\;m^{-2}\;y^{-1}$ lower in FD+ except in large areas of the high latitudes of the northern hemisphere, where it is up to approximately 0.1 $\mathrm{kgC}\;m^{-2}\;y^{-1}$ higher. For the S- and R-PFTs, GPP is lower by up to approximately 0.25 (S-PFTs) and 0.7 (R-PFTs) $\mathrm{kgC}\;m^{-2}\;y^{-1}$ in FD+. While it can also be higher for the S-PFTs (up to 0.1 $\mathrm{kgC}\;m^{-2}\;y^{-1}$), for the R-PFTs, we did not find a substantial increase (values < 0.1 $\mathrm{kgC}\;m^{-2}\;y^{-1}$). The described patterns can emerge for two reasons. First, the PFT remains dominant but its resource access is limited by competitors in FD+. Second, in many areas the dominant PFT changes over time. A PFT that was dominant historically may lose its competitive advantage and decline in FD+ by the end of the century (see also Fig. 4). Still, because of the shifts in community composition until the end of the century, the disadvantage of this PFT in the community (FD+) is compensated by other PFTs.

### Representative Example Regions.

To highlight specific spatial features and prominent differences between scenarios, we present relative changes for GPP and SOC and the change in number of PFTs for three exemplary regions (*SI Appendix*, *Representative Example Regions*, Fig. S11, and Table S3): Uganda and South Sudan (Africa), Germany (Europe), and the Argentinian Pampas (Argentina, South America). This selection encompasses a gradient from tropical to temperate climates and serves to illustrate how community composition shifts along this climatic gradient. We selected these regions primarily to represent different climate zones rather than specific grassland systems. However, the regions also span different grassland systems (tropical savanna, temperate meadow, subtropical grassland), providing broader ecological contrast. GPP is lower in FD− in Uganda and South Sudan, Germany, and large parts of the Argentinian Pampas. The lower GPP in FD− is related to a loss of subordinate PFTs that cannot be compensated in Uganda and South Sudan, to a loss in productivity of the dominant PFT in Germany. For the Argentinian Pampas the model simulates shifts in the shares of C3 and C4 PFTs in FD+.

In Uganda and South Sudan, differences between FD+ and FD− are a result of the interactions between C4 PFTs. In areas with higher GPP and SOC in FD−, removal of competitors allows the C-PFT utilize resources more efficiently while in the remaining areas the removal of subordinate PFTs cannot be compensated by the dominant PFT (*SI Appendix*, *Uganda and South Sudan, Africa*).

While the magnitude of relative changes between FD+ and FD− is similar in Germany, the underlying reason is different. Spatial patterns of the differences in GPP and SOC between FD+ and FD− correspond to the number of PFTs removed in FD−. In parts of Germany, bioclimatic limits of the temperate and polar PFTs overlap and productivity loss is correlated to the number of PFTs removed. While the overlap of bioclimatic limits is debatable and should be resolved, the example shows how climate change may lead to a loss of productivity if PFTs that become increasingly unsuitable under climate change cannot be replaced (*SI Appendix*, *Germany, Europe*).

In the Argentinian Pampas, increasing temperatures lead to a replacement of C3 PFTs by C4 PFTs in FD+ because the differences in the photosynthesis module (*SI Appendix*, *Photosynthesis Module*) give the C4 PFTs an advantage. However, in some areas of the central Argentinian Pampas the more competitive C3 grass becomes more advantageous. Adjustments of the balance between C3 and C4 PFTs stabilize productivity of the community (*SI Appendix*, *Argentinian Pampas, South America*).

## Discussion

We were able to demonstrate the role of functional diversity for the GPP and SOC stocks of managed grasslands at the global scale, making use of spatially explicit simulations of two functional diversity scenarios with LPJmL-CSR. Generally, we find the direction of change and the order of magnitude plausible. The model was able to match observational data well (*SI Appendix*, *Evaluation of Model Performance*), despite our highly aggregated representation of functional diversity using CSR theory. Furthermore, projected changes in community composition are rooted in important trade-offs between ecological strategies. This is an approach with strengths and weaknesses regarding the LPJmL model in general, the CSR implementation, and its comparison to field experiments and ecological theory.

While LPJmL-CSR mechanistically represents competition for light, water, and nitrogen at the process level, we applied the CSR framework such that each strategy maintains consistent performance across all resource types. For example, Competitors excel under all resource limitations rather than specializing in competition for specific resources. This is a deliberate simplification to limit the number of parameters requiring calibration.

### Highlights and Limits of LPJmL-CSR.

LPJmL-CSR is a state of the art dynamic global vegetation model that includes a representation of functional diversity of managed grasslands and neither follows an individual-based approach nor represents entire trait distributions. While LPJmL-CSR has previously been applied at the local scale (34), we successfully calibrated it for a global application using only the three main CSR strategies. This is a major step forward. However, our evaluation highlighted the need for future model development. The representation of trade-offs between the different strategies is not sufficient to fully reproduce observational data (*SI Appendix*, *Evaluation of Model Performance*). Our comparison showed that the R-PFT is generally at a disadvantage and underrepresented compared to the data, while the C-PFT is generally at an advantage and therefore overrepresented.

This is likely because the current version of LPJmL-CSR lacks a key feature of R strategies, the representation of reproduction and mortality (34). While LPJmL-CSR simulates a higher establishment rate—representative of more offspring—for the R-PFT, reproduction costs are not differentiated between strategies. However, the R strategy is characterized by a large number of offspring (42) and lower reproductive cost which correlate with plant size (43). Additionally, the R strategy is successful in areas with a short growing season. In these areas, the other strategies cannot survive due to factors like drought or cold mortality (42). These mortality types are not represented in LPJmL-CSR. Implementing additional processes to close these gaps will likely improve simulation results. To represent seed banks, the approach of the QUINCY model which includes the allocation of resources to fruits and a seed bed might serve as an inspiration (44). Different mortality types could be implemented following existing modeling approaches. For example, the FATES model represents frost death based on temperature and PFT-specific parameters (45). Furthermore, we used only the three main strategies defined by the CSR theory. Simulated communities can follow intermediate strategies only if they are mixtures of the main strategies (PFTs). An alternative approach would implement intermediate strategies at the PFT level. For example, a CS-PFT could be added with traits lying between the C- and S-PFT. While this could improve model results further, it would also increase model complexity and, in turn, computation costs, necessary calibration efforts, and data requirements.

Comparing historical GPP and projections for SSP 1-2.6 and SSP 3-7.0 showed an increase of grassland GPP under climate change. Higher atmospheric ${\mathrm{CO}}_{2}$ concentrations and temperature facilitate productivity. However, increased water limitation should offset these effects due to reduced precipitation and increasing drought events (46). We suspect that the simulations underestimate water limitation and drought effects on vegetation because several related damage processes are not explicitly implemented in the model. Plant damage from drought and drought-induced mortality (such as from embolism) are not represented in LPJmL. This enables the PFTs to survive even severe droughts with only limited biomass loss through above- and below-ground litter production and tissue turnover. As a result, the amount of living biomass available for photosynthesis is overestimated at the end of a dry period.

While additional model development will likely improve the fit to data, the existence of data on community share of the different strategies limit the calibration process and therefore the performance of the calibrated model substantially.

The role of natural and controlled or accidental anthropogenic fires for grassland dynamics was not considered in this study. This poses another limitation worth noting because fires are important to remove moribund tissue, for nutrient cycling and as a trigger of new tissue growth (47–49). Future studies can make use of parallel model development that allow the simulation of natural and accidental anthropogenic (50) as well as controlled fires (51).

Another important limitation is the livestock management. We use a dataset that provides spatially explicit but temporally constant livestock densities. Dry matter intake depends on the amount and quality of available biomass, which is affected by the plant community composition (*SI Appendix*, *Livestock Grazing* and ref. 52). However, in reality, stocking densities can be adjusted to biomass availability. This adds an additional layer of complexity and potential for adaptation that require additional model development.

All simulations were conducted with a 0.5^°^ × 0.5^°^ resolution neglecting substantial heterogeneity within grid cells. Although LPJmL-CSR could be run at a higher resolution, respective input data, especially for management, are rare and climate data are already downscaled to 0.5^°^ × resolution from the coarser original resolution. Future studies might therefore address regions of specific interest and with appropriate data availability to provide insights into the role of functional diversity potentially under management change and fire in specific areas with subregional heterogeneity in growing conditions.

### The Role of Functional Diversity.

We used data from ref. 53 which provide a global map with almost full coverage for several plant traits combining TRY data and citizen science observations from iNaturalist. These data consist of photographs of plants taken by citizens and may contain a selection bias for rare or appealing species (54). Resulting trait ranges might therefore not be representative of typical managed grassland species. We added another layer of preprocessing by applying the StrateFy approach (55) to calculate C, S, and R shares of a community based on three plant traits. These multiple layers of processing are a source of uncertainty regarding the resulting community compositions. In the future, this approach could be refined by incorporating additional data sources. For example, sPlot-based traits maps from ref. 53 could improve representativeness. sPlot is a database of scientific experiments, so derived trait values are likely more representative of actual communities than citizen science data. However, sPlot does not cover the same spatial extent as iNaturalist and is insufficient alone for a global evaluation. A hybrid approach using sPlot data for bias correction of the iNaturalist-based product could be valuable in future research. Similar to the community share data, the GPP data are a remote sensing product that contains uncertainty as is evidenced when comparing different GPP datasets (56).

In line with the diversity productivity theory (57–59), LPJmL-CSR simulated substantially lower GPP and SOC stocks if the nondominant PFTs were removed. While this approach does not represent actual diversity loss, it can serve as a proxy to represent a very low biodiversity scenario comparable to the artificial communities of biodiversity experiments. Qualitatively, our results are in line with these experiments which show an increase in productivity with increasing species numbers (e.g., refs. 12 and 60). However, these experiments have been criticized for not being representative of natural systems. The artificial removal of diversity through weeding affects soil fertility (60). This in turn led to artificially low productivity in low-diversity plots (61). In fact, other studies found a maximum productivity at low diversity levels (62). In LPJmL-CSR, the loss of productivity is less severe because the model does not include pests, diseases, and disturbances through repeated weeding. As such, the FD− scenario may be more comparable to the loss of some, but not all, functional diversity.

Our results show an increase in the productivity of the dominant PFT in FD− compared to FD+ due to increased resource availability and reduced competition. However, the communities with low functional diversity (FD−) rarely obtained a higher productivity than the functionally diverse communities (FD+). The differences between FD+ and FD− in our example regions highlight that the underlying reasons may differ. In large parts of Uganda and South Sudan and Germany, the increase in productivity of the dominant PFT in FD− could not compensate the loss of productivity from nondominant PFTs. Only in communities where the dominant PFT could strongly increase its community share and therefore resource uptake, the increase in productivity of the dominant strategy could compensate the effect of functional diversity. This aligns with recent findings that the diversity productivity relationship is mediated by various factors such as climate, soil properties, management, or landscape structures (e.g., refs. 63–66). To provide an illustrative example, in a typical community of a productive grassland in Germany the majority of the productivity can be attributed to very few species such as perennial ryegrass (*Lolium perenne*). However, the species pool usually contains several subordinate and marginal species—e.g., Kentucky bluegrass (*Poa pratensis*) or Timothy (*Phleum pratense*) that can increase their share if the perennial ryegrass suffers under changing conditions.

When comparing SSP 1-2.6 and SSP 3-7.0 stronger differences between FD+ and FD− emerged in SSP 3-7.0 in areas where temperatures were at the edges of bioclimatic limits of some PFTs. This indicates that not only the compositional changes of already established PFTs are important for GPP and SOC but also the establishment of additional PFTs. However, LPJmL-CSR might overestimate the adaptation capacity of communities because migration occurs instantly and is not limited by distance. In the south-east of Germany, FD− showed lower productivity because the historically dominant PFT became unsuitable in the future climate leading to breakdown of the community. This shows that functional diversity can serve as an insurance against climate change, which was also shown in multiple field experiments (e.g., ref. 67). In North and South Carolina, however, the lower productivity in FD− was caused by the community’s lack of adaptability to climate change. The temperature increase led to an advantage of tropical C4 PFTs not present in 2014 when the dominant PFT for FD− was determined, but present in 2100 in FD+. This advantage is underpinned by the different photosynthesis mechanism of C3 and C4 plants that are included in LPJmL (*SI Appendix*, *Photosynthesis Module*). These results are in line with field experiments and empirical modeling for North America showing an advantage of C4 species at higher temperatures and predicting an increase of C4 species in areas where C3 and C4 species are both abundant (68, 69). This serves as an example how migration of plant species may help to adapt to climate change and lessen its impacts. While this is well researched (e.g., ref. 70), the velocity of climate change and species migration are not the same, limiting the natural adaptation capacity of communities (71, 72), which is not represented in LPJmL-CSR. Therefore, the effect of community adaptation simulated by LPJmL-CSR can only be interpreted as a potential effect.

## Conclusions

Using the LPJmL-CSR dynamic global vegetation model, we conducted a global-scale assessment of the role of functional diversity for GPP and SOC stocks of managed grasslands. In this first analysis, we compare two states: a scenario with functional diversity (FD+) to a scenario without functional diversity (FD−) quantifying the differences in GPP and SOC storage. However, the change in functional diversity is not a rapid state shift but long-term transition responding to climate change. In combination, climate change and functional adaptation of communities may encompass feedbacks and affect each other. For example, climate change is likely to outpace the natural adaptation capacity of managed grassland communities, accelerating the loss of functional diversity (73), which may affect the land carbon sink via reduced SOC stocks (6) and in turn the velocity of atmospheric ${\mathrm{CO}}_{2}$ increase. While we see opportunities for further model improvement, our results are generally in line with observations and ecological theory. Therefore, LPJmL-CSR could serve as a tool to further investigate aforementioned interactions at the global scale.

Furthermore, while our results are limited by model constraints and should only be extrapolated with caution to new conditions, they clearly show once more that preventing the loss of functional diversity will help to maintain grassland productivity under climate change, which is crucial for many livelihoods, especially in the global south (74, 75). The loss of SOC storage that goes hand in hand with functional diversity loss reduces the terrestrial carbon sink, negatively affecting the remaining carbon budget. This is especially important considering that already today, grassland degradation and overgrazing lead to significant carbon losses which are likely to increase under projected climate change impacts. LPJmL-CSR could be used to assess how functional diversity responds to varying management intensities and land degradation globally, and how these dynamics feed back to other Earth system components. Our findings underpin the need for development and application of grassland management practices that support functional diversity, continued research on breeding to compensate climate change impacts and to incentivize policy-makers to assist farmers in the necessary transition.

## Materials and Methods

LPJmL-CSR has only been applied at the local scale so far (34) and global-scale simulations require a calibration.

### LPJmL-CSR.

A detailed description of LPJmL is provided in previously published work (27, 28, 30, 34, 52). Briefly summarized, LPJmL-CSR simulates the full carbon, water, and nitrogen cycles of managed grasslands using a Farquhar-based photosynthesis approach (76) that explicitly accounts for the differences between C3 and C4 photosynthesis pathways (see *SI Appendix*, *Photosynthesis Module* as well as ref. 27 and references therein) at daily time steps. Assimilated carbon is subsequently reduced by autotrophic respiration and distributed between plant organs. Above- and belowground biomass is then transferred to litter pools through tissue turnover. Finally, to determine grazed biomass, we use a livestock module that calculates dry matter intake based on livestock density and forage quality (see *SI Appendix*, *Livestock-Grazing* and ref. 52). The representation of managed grasslands does currently not include any tree cover ignoring tree grass interaction in biomes such as savannas. A novelty in LPJmL-CSR is the distinction between different ecological strategies based on CSR theory, which requires an improved representation of competition for resources and trade-offs between strategies (*SI Appendix*, *Overview of LPJmL-CSR* and ref. 34). The grassland PFTs differ greatly between LPJmL and LPJmL-CSR: in LPJmL, managed grasslands are represented using three grass PFTs (27, 28). These three PFTs consist of a polar, a temperate, and a tropical PFT. LPJmL-CSR allows the introduction of additional grass PFTs (34). For this study, we calibrated parameters for three tropical, three temperate, and three polar grass sub-PFTs based on the respective original LPJmL PFTs.

### Calibration and Evaluation Data.

**Fig. 5.**
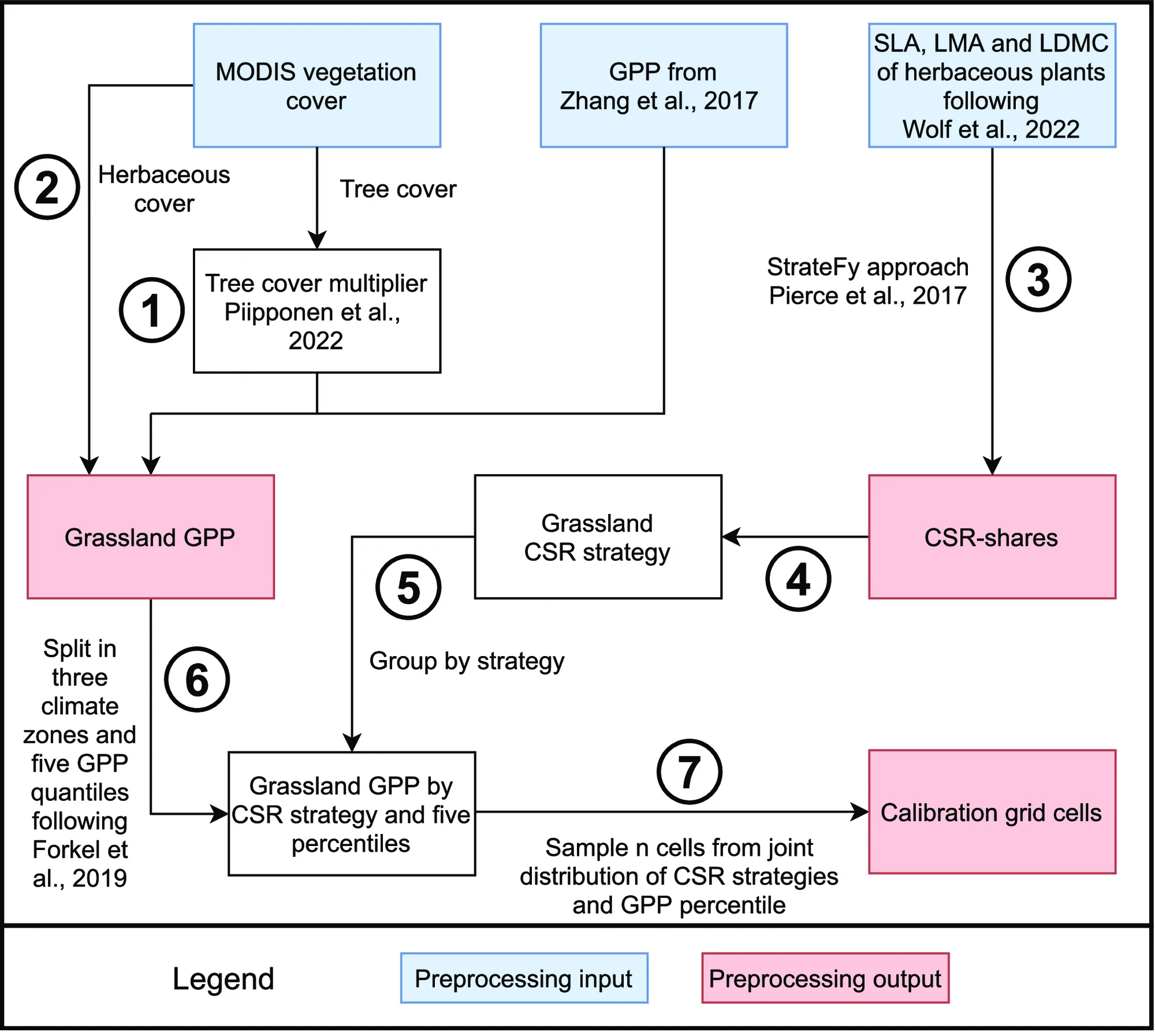
Overview of processing steps to prepare the calibration data and selection of grid cell subset for calibration. All data have a global coverage and a $0.05^{{}^{\circ}}$ by $0.05^{{}^{\circ}}$ resolution. Colored boxes highlight preprocessing input (blue), temporal data of intermediate steps (white), and output data (red). Arrows show the information flow from input to output data and numbers reference specific steps described in *Materials and Methods*.

We calibrated the model using global data on grassland GPP and the community share of C-, S-, and R-PFTs, which had to be processed. Fig. 5 shows an overview of all preprocessing steps. Each step is marked with a number used for reference within the text.

#### GPP.

We used a publicly available global remote sensing product for GPP (77). This product contains total monthly GPP data ($GPP_{\mathrm{tot}}$) from 2000 to 2016 for $0.05^{{}^{\circ}}\times 0.05^{{}^{\circ}}$ grid cells. To obtain grassland GPP ($GPP_{\mathrm{herb}}$) we had to account for tree cover and scaled the resulting values to the grassland fraction for each grid cell (Eq. **1** and Fig. 5, step 1).

[1] $$\begin{array}{c} GPP_{\mathrm{herb}}=GPP_{\mathrm{tot}}\cdot s_{\mathrm{tree}}/f_{\mathrm{grassland}}. \end{array}$$

$s_{\mathrm{tree}}$ is a tree cover multiplier (Eq. **2**), calculated from annual MODIS MCD12C1 tree cover data from 2001 to 2016 at a spatial resolution of $0.05^{{}^{\circ}}$ by $0.05^{{}^{\circ}}$ (78) following the approach of ref. 79.

[2] $$\begin{array}{c} s_{\mathrm{tree}}=exp\left(-4.45521\cdot f_{\mathrm{tree}}\right). \end{array}$$

Eq. **2** is an empirical relationship between tree cover ($f_{\mathrm{tree}}$) and the total productivity of this tree cover. It is used to account for the different produc tivity levels of woody and herbaceous vegetation. $f_{\mathrm{grassland}}$ is the fraction of the area not covered by trees from the same product and is used to scale herbaceous GPP to the entire grid cell to make it compatible with LPJmL (Fig. 5, step 2). We used the annual data to scale each monthly GPP value. Because data for 2000 were not available within MODIS MCD12C1, we assumed the tree cover of 2001 for 2000.

Afterward, we aggregated $GPP_{\mathrm{herb}}$ to $0.5^{{}^{\circ}}$ by $0.5^{{}^{\circ}}$ because this is the resolution at which LPJmL-CSR was run. We weighted each $0.05^{{}^{\circ}}$ by $0.05^{{}^{\circ}}$ grid cell using its area fraction of the $0.5^{{}^{\circ}}$ by $0.5^{{}^{\circ}}$ grid cell (Eq. **3**).

[3] $$\begin{array}{c} GPP_{herb,0.5,i}=\sum_{j=1}^{100}w_{j}\cdot GPP_{herb,j}, \end{array}$$

with $w_{j}=A_{j}/A_{i}$. *SI Appendix*, Fig. S2 shows the time series average $GPP_{\mathrm{herb}}$ and the 5th to 95th percentile range.

#### Community share of the C-, S-, and R-PFTs.

To obtain the community share for C-, S-, and R-PFTs, we combined global traits maps of specific leaf area, leaf mass per area and leaf dry matter content at a spatial resolution of $0.5^{{}^{\circ}}$ by $0.5^{{}^{\circ}}$ obtained from ref. 53 with the StrateFy approach (55, Fig. 5, step 3). The StrateFy approach is described in ref. 55 and can be used to calculate the C, S, and R shares of a species or community from specific leaf area, leaf mass per area and leaf dry matter content data. Pierce et al. 55 developed the StrateFy approach using TRY data (80) which also provided the basis for the trait estimation of the algorithm used by Wolf et al. 53. Global trait maps are obtained by combining citizen science data from iNaturalist provided by the global biodiversity information facility (GBIF) database (81), with trait data from TRY using a convolutional neural networks approach (53, 54). The convolutional neural networks are trained to recognize plant traits from morphological features present in photographic images of plants. The approach is described in detail in ref. 54. *SI Appendix*, Fig. S3 shows the C, S, and R share for each grid cell obtained using the median, average, 5th or 95th percentile trait estimates. We assumed the median estimates to represent the share of the C, S, and R strategy. In LPJmL-CSR the community share is represented using the foliage projective cover.

### Model Calibration and Evaluation.

We calibrated LPJmL-CSR against the GPP and the community share of the C-, S-, and R-PFT. For both variables we calculate a cost using simulation results from 2000 to 2016, matching the time period of the GPP data. Because community share of the C-, S-, and R-PFT data is only available for one year, we use the 2000 to 2016 average.

We calibrated LPJmL-CSR using a genetic optimization (GENOUD, 82) algorithm which is the basis for a model-data integration (MDI) approach for LPJmL implemented in the LPJmLMDI R package (83). The approach is described in more detail in refs. 83 and 84. LPJmLMDI calculates a cost based on the Kling–Gupta efficiency (85) for each calibration variable *d* to evaluate the model fit compared to observations.

[4] $$\begin{array}{c} Cost=\sqrt{\sum_{d=1}^{N}\left({\left(\frac{\bar{s_{d}}}{\bar{o_{d}}}-1\right)}^{2}+{\left(\frac{\sigma_{s,d}}{\sigma_{o,d}}-1\right)}^{2}+{\left(r\left(s_{d},o_{d}\right)-1\right)}^{2}\right)}. \end{array}$$

$\bar{s}$ and $\bar{o}$ are the spatial (grid cells) and temporal (years) mean values of the simulations *s* and the observations *o*, respectively. These represent the bias component in the Kling–Gupta efficiency computation. The variance component is represented by *σ*, which is the variance. Finally, the correlation component is represented by r, which is the Pearson correlation coefficient (both over space and time) (83). Each of the components was weighted by the uncertainty of the observations to account for spatiotemporal uncertainty in the cost function. The algorithm is initialized by sampling a preset number (generation) of individual parameters sets (individuals) from a multivariate normal distribution reflecting the parameter space. After each individual parameter set has been evaluated, a new generation is initialized considering the divergence between simulation and observations of the previous generations. For details of how GENOUD derives the parameter sets for generations following the initial generation we refer to ref. 86. For each climate zone (tropical, temperate, polar), we sampled 64 grid cells from the joined distribution of grassland GPP and CSR strategy (Fig. 5, step 4 to 7). Climate zones were defined following ref. 87 grouping frigid and cold temperate to boreal, subtropical, and tropical to tropical and the remainder to temperate. First, we grouped the community shares of the C-, S-, and R-PFTs into 19 strategies (C, C/CR, C/CS, CR, C/CSR, CS, CR/CSR, CS/CSR, R/CR, CSR, S/CS, R/CSR, S/CSR, R, SR/CSR, S, R/SR, S/SR, and SR), which are based on the thresholds for C, S, and R shares defined by Pierce et al. (55), Fig. 5, step 4. These 19 strategies are only used in the preprocessing to select grid cells that are representative of the range of strategies observed. In LPJmL-CSR intermediate strategies are reflected through shares of multiple PFTs (e.g., CS strategy is represented by proportional shares of the C- and an S-PFT and not through a CS-PFT) Second, we assign each GPP value the respective CSR strategy derived in Fig. 5, step 4 (Fig. 5, step 5). Third, we divide the GPP data into five equidistant intervals based on the level of productivity (very low, low, medium, high, very high) for each of the three climate zones (tropical, temperate, and polar) (Fig. 5, step 6). Finally, we sample 64 grid cells from each climate zone (Fig. 5, step 7) to represent the distribution of GPP and CSR-shares. We sample from all grid cells for the calibration. In contrast, we subset the data to only contain grid cells where the data on historical grassland fractions by Ostberg et al. 88 contain managed rangeland and grassland according to Allen et al. 89 for the evaluation and our scenario analysis. This results in a broader distribution of GPP and community shares of the C-, S-, and R-PFTs for the sampled grid cells and maximizes the domain to which the calibration results are applicable. We included 16 PFT-specific parameters that relate to the difference between CSR strategies to calibrate three PFTs per climate zone (see *SI Appendix*, Table S2 and *Overview of LPJmL-CSR* for parameter descriptions). The selected parameters distinguish C-, S-, and R-PFTs through their role in various processes that determine competitive success for light, water, and mineral nitrogen, their reproductive strategy and life cycle, grazing avoidance or tolerance, and biological nitrogen fixation. The parameters and their ranges were selected based on past sensitivity analysis (84, 90), publications showing their importance for biomass dynamics (30, 34) and expert assessment. Parameter selection prioritized those governing resource acquisition strategies (light interception via SLA and LAI, water access via root allocation, nitrogen uptake), stress tolerance mechanisms (tissue longevity, transpiration), and reproductive allocation, as these processes most strongly differentiate C, S, and R strategies in empirical and theoretical frameworks (31). The calibration of each climate zone comprised approximately 15 generations consisting of up to 2,000 individuals each.

A subset of the data of the remaining grid cells that were not used for the calibration were used for the evaluation. This subset contains only grid cells in which the data on historical grassland fractions by Ostberg et al. 88 contain managed grassland in at least one of the years used for calibration (2000 to 2016). In total, community share data for 28,767 grid cells and GPP data for 37,742 grid cells for 204 time steps (17 y × 12 mo) were used for the evaluation. For the evaluation, we used the differences between simulated and observed GPP and foliage projective cover. For GPP, we evaluate the model against the monthly data and annual sums but do not aggregate the data further. Similar to the calculation of the cost function, we average the community share of the C-, S-, and R-PFT simulation results from 2000 to 2016 and compare these against the observations.

We did not calibrate and evaluate SOC stocks. However, in LPJmL, these are to a large amount determined by the inputs into the litter layer and the soil that are driven by plant productivity. Therefore, model performance regarding GPP and SOC stocks is related and we are confident in our analysis of SOC stocks.

### Modeling Protocol.

For the calibration and evaluation simulations we used the climate data from the GSWP3-W5E5 dataset (91–93), historical atmospheric ${\mathrm{NO}}_{3}^{-}$ and ${\mathrm{NH}}_{4}^{+}$ deposition (94), historical atmospheric CO_2_ concentrations (95) and grazing management data (96).

We used the optimized parameters to simulate two different functional diversity scenarios using climate data from five general circulation models GFDL-ESM4 (97), IPSL-CM6A-LR (98), MPI-ESM1-2-HR (99), MRI-ESM2-0 (100) and UKESM1-0-LL (101)] and two representative forcing scenarios provided by the intersectoral impact model intercomparison project (ISIMIP, 93). The climate data have a daily temporal resolution and spatial resolution of $0.5^{{}^{\circ}}$ by $0.5^{{}^{\circ}}$ and include average, minimum and maximum temperature, precipitation, downward short wave radiation, long wave net radiation and wind speed. Data on ${\mathrm{CO}}_{2}$ concentration as well as deposition of ${\mathrm{NO}}_{3}^{-}$ and ${\mathrm{NH}}_{4}^{+}$ were also available from ISIMIP but not per general circulation model. All ISIMIP datasets distinguish a historical period from 1850 to 2014 and a future period from 2015 to 2100, for which simulated data were bias-corrected to observational data products. Additionally, we used data on historical grassland fractions (88) and grazing management data (102). Grassland fractions were only available until 2017 and we assumed the fractions to be constant for future periods using the values for 2014, i.e., the last year of the historical period. Grazing management assumes a daily grazing with a fixed livestock density per grid cell (i.e., constant feed energy demand) for the entire simulation period. The livestock densities are used to determine the dry matter intake depending on forage quality (see *SI Appendix*, *Livestock Grazing* and ref. 52). These assumptions allow us to isolate the effect of climate change and eliminate other factors that influence global output variables such as grassland expansion or reduction as well as the impacts of management change.

For all scenarios, we conducted two spin-up simulations of 3,500 y with natural vegetation only and a subsequent spin-up of 420 y, gradually introducing historical land use patterns (88). During the spin-up, climatic forcing was a random permutation of the climate data from 1901 to 1930. The first spin-up simulation ensures that the C and N balances are in an equilibrium before the beginning of anthropogenic land-use change. The second spin-up simulation captures the effects of historical land-use change on the C and N cycle. Following the two spin-up simulations, the model is run from 1850 until 2100 using the transient input data.

During the historical period, we allow all PFTs to be established so that the community composition can evolve dependent upon environmental conditions and management. For the future period, we simulate the two scenarios described in the introduction. In FD−, the PFTs with the highest foliage projective cover at the end of the historical period in 2014 were selected as the dominant PFTs for each 0.5^°^ grid cell.

## Supplementary Material

Appendix 01 (PDF)

## Data Availability

Partial model output data, model code, and scripts to run the model and visualizations have been deposited in Zenodo: Code and data for Global grassland productivity and carbon storage benefit from functional diversity already under moderate climate change (https://doi.org/10.5281/zenodo.18152694) (103). Previously published data were used for this work. The data and code to produce figures for the other general circulation models are available (41).
