# Supplementary material for: Global grassland productivity and carbon storage benefit from functional diversity under climate change
Source: Proc Natl Acad Sci U S A. 2026 Oct 7;123(41):e2528067123. doi: 10.1073/pnas.2528067123 (PMC13663838; doi:10.1073/pnas.2528067123)
Supplement: Supplementary file 1 [file pnas.2528067123.sapp.pdf]

Supporting Information for

Global grassland productivity and carbon storage benefit from functional diversity already under moderate climate change

Stephen Björn Wirth, Christoph Müller, Friedhelm Taube, Jens Heinke, Britta Tietjen and Susanne Rolinski

Stephen Wirth.
E-mail: stephen.wirth@pik-potsdam.de

This PDF file includes:

Supporting text
Figs. S1 to S20
Tables S1 to S3
SI References

Supporting Information Text

LPJmL. LPJmL simulates the terrestrial carbon and nitrogen cycles of natural and managed vegetation. The daily computation of the gross primary production (GPP) from photosynthesis (see Photosynthesis) is at the core of the model. This computation accounts for the co-limitation of temperature, radiation, water and nutrient availability. Natural vegetation is represented by eight tree and three grass PFTs. These PFTs are allowed to be established within predefined bioclimatic limits to ensure that, for example, tropical PFTs do not grow in polar climates. Due to overlap of the bioclimatic limits, up to six PFTs can be established per grid cell. We are aware, that the resulting number of PFTs that are implemented in LPJmL-CSR are a very coarse representation of functional diversity which should be kept in mind with respect to our results. However, the representation of different ecological strategies is a large improvement towards a better representation of functional diversity compared to other dynamic global vegetation models and also other versions of LPJmL. Together, the established PFTs form communities that differ in their functional diversity. We use the presence of different strategies and their community share to infer information on the role of functional diversity. If only one strategy is present, the functional diversity is low. In contrast, if all strategies are present, the functional diversity is high. When multiple strategies are present but one is strongly dominant, functional diversity is less important at that point in time. However, if multiple strategies are present, community composition may adapt over time, making functional diversity important for the long-term performance of the community. In our simulation experiments (see Modelling protocol), we aim to quantify such effects by artificially reducing functional diversity.

Photosynthesis module. LPJmL simulates photosynthesis using a "big leaf" representation of the Farquhar approach (1), adjusted for global-scale models (2, 3), following the strong optimality hypothesis (4). The approach accounts for the different behaviour of C3 and C4 photosynthesis with respect to CO_2 fixation and adaptation to climate, which reflects the advantage of C4 plants having lower transpiration than C3 plants in hotter and drier climates. The model specifically accounts for the different intrinsic quantum efficiencies for CO2 uptake of C3 and C4 plants (α_{path}) and leaf internal to ambient CO_2 pressure ratios (4–6). While we did not use any parameters involved in the inner workings of photosynthesis in our calibration, they are important for the interactions between C3 and C4 PFTs. Table SI S1 shows all parameters that differ between C3 and C4 PFTs, that play a role in photosynthesis and competition, and that were not explicitly calibrated for this study. The leaf respiration (b) and autotrophic respiration rate (r_{PFT}) parameters control respiratory losses to determine NPP (4). β_{leaf} is the leaf albedo parameter and part of the determination of absorbed photosynthetically active radiation (PAR). The temperature limits either constrain photosynthesis or plant establishment. $T_{lim,CO_2,low}$ and $T_{lim,CO_2,high}$ determine the temperature inhibition of CO_2 uptake. $T_{lim,opt,photo,low}$ and $T_{lim,opt,photo,high}$ represent the temperature range for optimal photosynthesis. $T_{lim,cold,month,low}$ and $T_{lim,cold,month,high}$ are bioclimatic limits constraining tropical, temperate and polar PFTs to their respective climate zones. The phenology parameters are used in the logistic functions to determine the growing season index described in detail in (7). For managed grasslands, only day-to-day differences are used to adjust allocation between leaves and roots to increase allocation to leaves at the beginning of the growing season (8). $C : N_{leaf,min}$, $C : N_{leaf,median}$ and $C : N_{leaf,max}$ are the allowed ranges in which the leaf C:N ratio can vary and control plant N demand, uptake as well as allocation of C and N if minimum or maximum values are exceeded.(9).

Biological nitrogen fixation. LPJmL simulates biological nitrogen fixation as described in (10). If nitrogen uptake from the soil is insufficient to meet the PFT's nitrogen demand, the PFT can fix additional nitrogen. The amount of additional fixation has several constraints: Soil temperature and soil water status determine the potential amount that can be fixed. However, actual fixation can never exceed the actual demand and depends on the amount of NPP the PFT can allocate to biological nitrogen fixation and the maximum fixer fraction of the climate zone. In theory, this approach could be used to simulate leguminous (fixer fraction of one) and non-leguminous (fixer fraction of zero) PFTs separately. However, the current model version uses one PFT that represents a hybrid of both. In practice, individual PFTs act more like nitrogen fixers when nitrogen-limited and less so when nitrogen is abundant.

Grass PFTs. In LPJmL and LPJmL-CSR, grass PFTs are represented using leaf and root biomass stocks. The following daily processes determine the vegetation and soil biomass stocks of managed grasslands: (i) Carbon assimilation from photosynthesis to obtain GPP (see Photosynthesis), (ii) autotrophic respiration to obtain the net primary productivity (NPP), (iii) transfer of leaf and root tissue to the litter layer, (iv) allocation of NPP to leaf and root biomass stocks and potentially (v) livestock grazing (see (11)) or mowing. The amount of photosynthesis, litter fall and grazing or harvest is determined depending on PFT-specific parameters – which are inferred from functional traits, environmental conditions and management. Resource availability drives competition which in turn determines the community composition during each time step. A full overview of PFT-specific parameters is given in the studies listed above. For LPJmL-CSR several parameters (Tab. SI 1) were adjusted during calibration (see Model calibration and evaluation).

Overview of LPJmL-CSR. LPJmL-CSR contains several unique features to enable the representation of CSR-PFTs. First, resource distribution between PFTs was extended to separate access to aboveground and belowground resources. Aboveground, light is distributed between PFTs depending on their foliage projective cover, similar to the original LPJmL model. While in the original model water was also distributed depending on foliage projective cover, in LPJmL-CSR it is distributed based on the root network, allowing better niche differentiation. Second, LPJmL-CSR connects leaf longevity with SLA via the trade-off described in the fast-slow dimension of the leaf economic spectrum (12), which is a key feature to distinguish C- and R-PFTs from S-PFTs. Finally, while in the original model only one average individual is used to represent the grassland of an entire

Table S1. Parameter differences between C3 and C4 grass PFTs in LPJmL

Parameter	Tropical C4	Temperate C3	Polar C3	Unit
α_{path}	0.053	0.08	0.08	$[-]$
b	0.035	0.015	0.015	$[-]$
r_{PFT}	0.2	1.2	1.2	$[\mathrm{gC\ gN^{-1}\ day^{-1}}]$
β_{leaf}	0.21	0.23	0.23	$[-]$
Temperature parameters				
$T_{lim,CO_2,low}$	6.0	-4.0	-4.0	$[^\circ\mathrm{C}]$
$T_{lim,CO_2,high}$	55.0	45.0	38.0	$[^\circ\mathrm{C}]$
$T_{lim,opt,photo,low}$	20.0	10.0	10.0	$[^\circ\mathrm{C}]$
$T_{lim,opt,photo,high}$	45.0	30.0	25.0	$[^\circ\mathrm{C}]$
$T_{lim,cold,month,low}$	7.0	-39.0	-1000	$[^\circ\mathrm{C}]$
$T_{lim,cold,month,high}$	1000	15.5	-2.6	$[^\circ\mathrm{C}]$
Phenology: cold temperature				
sl_{cold}	0.91	0.31	0.13	$[-]$
$base_{cold}$	6.4	5.0	2.8	$[-]$
τ_{cold}	0.20	0.010	0.20	$[-]$
Phenology: warm temperature				
sl_{heat}	1.47	0.24	0.24	$[-]$
$base_{heat}$	29.2	32.0	26.1	$[-]$
τ_{heat}	0.20	0.20	0.20	$[-]$
Phenology: light				
sl_{light}	64.2	23.0	23.0	$[-]$
$base_{light}$	69.9	75.9	50.0	$[\mathrm{W\ m^{-2}}]$
τ_{light}	0.40	0.22	0.38	$[-]$
Phenology: water stress				
sl_{water}	0.10	0.52	0.88	$[-]$
$base_{water}$	41.7	53.1	1.0	$[\%]$
τ_{water}	0.17	0.010	0.94	$[-]$
Nitrogen				
$CN_{leaf,min}$	17.4	10.5	10.5	$[\mathrm{gC\ gN^{-1}}]$
$CN_{leaf,median}$	34.0	19.9	19.9	$[\mathrm{gC\ gN^{-1}}]$
$CN_{leaf,max}$	66.9	37.9	37.9	$[\mathrm{gC\ gN^{-1}}]$
Biological N fixation				
$T_{BNF,,opt,low}$	20	18	12	$[^\circ\mathrm{C}]$
$T_{BNF,,opt,high}$	35	35	25	$[^\circ\mathrm{C}]$

grid cell, LPJmL-CSR uses a dynamic representation of average individuals that depends on reproduction and mortality. This allows the model to distinguish different reproductive strategies. Specifically, R-PFTs invest more into reproduction to ensure their survival via offspring, while C- and S-PFTs invest more into tissue growth. While all details on the implementation are provided in (8), we briefly explain the role of the parameters in Tab. SI S2 for LPJmL-CSR. g_{min} is the minimum canopy conductance determining the minimum transpiration that occurs even in the absence of photosynthesis. The specific leaf area (SLA) is used to determine the leaf area index (LAI) and in turn light interception, but also leaf longevity and tissue turnover. $lmro$ is the target leaf-to-root mass ratio and controls the amount of carbon and nitrogen allocated to the respective organs. LAI_{sapl} is the leaf area index of a sapling, which is used to calculate the carbon and nitrogen pools of a sapling. Together with the establishment rate (k_est), which gives the increase in number of average individuals, LAI_{sapl} determines the change in the average individual's carbon and nitrogen pools from reproduction. E_{max} is the maximum transpiration rate and represents the water demand limitation (maximum amount of water uptake) of photosynthesis. The light extinction coefficient (k_{light}) is used to calculate the amount of light absorbed by the vegetation layer and is used in the calculation of foliage projective cover, therefore determining the distribution of light between PFTs. $mort_{max}$ is the maximum mortality rate. Currently, LPJmL-CSR does not distinguish different mortality types. Instead, mortality is determined using $mort_{max}$ and the ratio of daily biomass changes to leaf area. As a result, mortality increases with decreasing biomass gains, which was originally implemented to represent age-related mortality. However, because biomass change is small or even negative under unfavourable conditions this also leads to increased mortality. k_{root} is the belowground equivalent of k_{light} and serves as a proxy for the branching of the root network and the ability to compete for water in each soil layer. $V_{max,up}$ is the maximum uptake rate per unit fine root mass for mineral nitrogen from the soil. The same value is used for NO_3^- and NH_4^+. To calculate the actual uptake, the model accounts for limitations from temperature, mineral N availability, current plant N content and root distribution. β_{root} is used to calculate the distribution of roots across the soil layers following (13) and therefore controls the amount of water a plant can obtain from each soil layer. $C_{max,BNF}$ and $N_{max,BNF}$ are parameters used in the calculation of symbiotic nitrogen fixation (10). $C_{max,BNF}$ combines two constraints: the maximum amount of NPP that can be allocated to N fixation and the share of N fixers in the respective biome. $N_{max,BNF}$ is the potential N fixation rate, subsequently reduced to account for limitations from soil temperature and water availability and root distribution. k_{store} is used to increase N demand — which is initially calculated from RuBisCo and structural N demand — to create reserves.

Table S2. PFT-specific minimum, maximum, default and optimised values for calibrated parameters for the competitive (C), stress tolerant (S) and ruderal (R) PFTs for tropical (Tr), temperate (Te) and polaar (Po) climates. All PFTs are herbaceous (H). All parameters are explained in 'Overview of LPJmL-CSR' for parameter definitions before this table.

PFT	Parameter	Unit	Minimum	Maximum	Default	Optimum
C-TrH	g_{min}	$[\mathrm{mm\ s^{-1}}]$	0.7	2	0.8	1.15
C-TrH	SLA	$[\mathrm{m^2\ gC^{-1}}]$	0.2	0.4	0.3	0.377828
C-TrH	$lmro$	$[-]$	0.7	1.5	0.8	1.49
C-TrH	LAI_{sapl}	$[-]$	0.001	0.03	0.01	0.027
C-TrH	E_{max}	$[\mathrm{mm\ d^{-1}}]$	6	12	7	7.13
C-TrH	k_{light}	$[-]$	0.4	0.8	0.5	0.55
C-TrH	$mort_{max}$	$[\mathrm{Average\ individuals\ m^{-2}\ d^{-1}}]$	0.09	0.4	0.1	0.17
C-TrH	k_{est}	$[\mathrm{Average\ individuals\ m^{-2}\ d^{-1}}]$	300	3000	365	2604
C-TrH	k_{root}	$[-]$	0.005	0.025	0.01	0.0076
C-TrH	$V_{max,up}$	$[\mathrm{gN\ gC^{-1}\ d^{-1}}]$	4	6	5.51	5.844
C-TrH	β_{root}	$[-]$	0.9	0.98	0.972	0.928
C-TrH	$C_{max,BNF}$	$[-]$	0.004	0.08	0.03	0.038
C-TrH	k_{store}	$[-]$	0.05	0.3	0.05	0.11
C-TrH	$N_{max,BNF}$	$[\mathrm{gN\ m^{-2}\ d^{-1}}]$	0.01	1	0.5	0.97
C-TrH	f_{avoid}	$[-]$	0.5	1.5	1	1.38
S-TrH	g_{min}	$[\mathrm{mm\ s^{-1}}]$	0.3	1	0.8	0.36
S-TrH	SLA	$[\mathrm{m^2\ gC^{-1}}]$	0.01	0.2	0.0337936	0.19654
S-TrH	$lmro$	$[-]$	0.3	1	0.8	0.53
S-TrH	LAI_{sapl}	$[-]$	0.009	0.05	0.01	0.03
S-TrH	E_{max}	$[\mathrm{mm\ d^{-1}}]$	3.5	8	7	6.91
S-TrH	k_{light}	$[-]$	0.4	0.8	0.5	0.7
S-TrH	$mort_{max}$	$[\mathrm{Average\ individuals\ m^{-2}\ d^{-1}}]$	0.05	0.2	0.1	0.15
S-TrH	k_{est}	$[\mathrm{Average\ individuals\ m^{-2}\ d^{-1}}]$	300	3000	365	1807
S-TrH	k_{root}	$[-]$	0.005	0.025	0.01	0.023
S-TrH	$V_{max,up}$	$[\mathrm{gN\ gC^{-1}\ d^{-1}}]$	4	6	5.51	5.24
S-TrH	β_{root}	$[-]$	0.9	0.98	0.972	0.915
S-TrH	$C_{max,BNF}$	$[-]$	0.004	0.08	0.03	0.04
S-TrH	k_{store}	$[-]$	0.05	0.3	0.05	0.14
S-TrH	$N_{max,BNF}$	$[\mathrm{gN\ m^{-2}\ d^{-1}}]$	0.01	1	0.5	0.08
S-TrH	f_{avoid}	$[-]$	0.5	1.5	1	1.22
R-TrH	g_{min}	$[\mathrm{mm\ s^{-1}}]$	0.6	2	0.8	1.42
R-TrH	SLA	$[\mathrm{m^2\ gC^{-1}}]$	0.4	0.8	0.5	0.739233
R-TrH	$lmro$	$[-]$	0.3	1	0.8	0.9
R-TrH	LAI_{sapl}	$[-]$	0.005	0.02	0.01	0.016
R-TrH	E_{max}	$[\mathrm{mm\ d^{-1}}]$	6	12	7	11.67
R-TrH	k_{light}	$[-]$	0.2	0.5	0.4	0.3
R-TrH	$mort_{max}$	$[\mathrm{Average\ individuals\ m^{-2}\ d^{-1}}]$	0.05	1	0.2	0.19
R-TrH	k_{est}	$[\mathrm{Average\ individuals\ m^{-2}\ d^{-1}}]$	3000	6000	3650	3034
R-TrH	k_{root}	$[-]$	0.005	0.025	0.01	0.0091
R-TrH	$V_{max,up}$	$[\mathrm{gN\ gC^{-1}\ d^{-1}}]$	4	6	5.51	4.99
R-TrH	β_{root}	$[-]$	0.9	0.98	0.972	0.933
R-TrH	$C_{max,BNF}$	$[-]$	0.004	0.08	0.03	0.045
R-TrH	k_{store}	$[-]$	0.05	0.3	0.05	0.16
R-TrH	$N_{max,BNF}$	$[\mathrm{gN\ m^{-2}\ d^{-1}}]$	0.01	1	0.5	0.43
R-TrH	f_{avoid}	$[-]$	0.5	1.5	1	1.42
C-TeH	g_{min}	$[\mathrm{mm\ s^{-1}}]$	0.7	2	0.8	1.57
C-TeH	SLA	$[\mathrm{m^2\ gC^{-1}}]$	0.04	0.14	0.1	0.118976
C-TeH	$lmro$	$[-]$	0.7	1.5	0.8	1.3
C-TeH	LAI_{sapl}	$[-]$	0.001	0.05	0.01	0.034
C-TeH	E_{max}	$[\mathrm{mm\ d^{-1}}]$	6	12	7	8.69
C-TeH	k_{light}	$[-]$	0.4	0.8	0.5	0.52
C-TeH	$mort_{max}$	$[\mathrm{Average\ individuals\ m^{-2}\ d^{-1}}]$	0.09	0.4	0.1	0.29
C-TeH	k_{est}	$[\mathrm{Average\ individuals\ m^{-2}\ d^{-1}}]$	300	3000	365	2207
C-TeH	k_{root}	$[-]$	0.005	0.025	0.01	0.0084
C-TeH	$V_{max,up}$	$[\mathrm{gN\ gC^{-1}\ d^{-1}}]$	4	6	5.51	5.79
C-TeH	β_{root}	$[-]$	0.9	0.95	0.943	0.906
C-TeH	$C_{max,BNF}$	$[-]$	0.004	0.08	0.03	0.019
C-TeH	k_{store}	$[-]$	0.05	0.3	0.05	0.08
C-TeH	$N_{max,BNF}$	$[\mathrm{gN\ m^{-2}\ d^{-1}}]$	0.01	1	0.5	0.91
C-TeH	f_{avoid}	$[-]$	1	1.5	1	1.43

Table S2 (continued): PFT-specific minimum, maximum, default and optimised values for calibrated parameters for the competitive (C), stress tolerant (S) and ruderal (R) PFTs for tropical (Tr), temperate (Te) and polaar (Po) climates. All PFTs are herbaceous (H). All parameters are explained in 'Overview of LPJmL-CSR' for parameter definitions before this table.

PFT	Parameter	Unit	Min	Max	Def	Opt
S-TeH	g_{min}	$[\mathrm{mm\ s^{-1}}]$	0.3	1	0.8	0.48
S-TeH	SLA	$[\mathrm{m^2\ gC^{-1}}]$	0.01	0.04	0.0337936	0.010082
S-TeH	$lmro$	$[-]$	0.3	1	0.8	0.39
S-TeH	LAI_{sapl}	$[-]$	0.009	0.05	0.01	0.035
S-TeH	E_{max}	$[\mathrm{mm\ d^{-1}}]$	3.5	8	7	4.13
S-TeH	k_{light}	$[-]$	0.4	0.7	0.5	0.43
S-TeH	$mort_{max}$	$[\mathrm{Average\ individuals\ m^{-2}\ d^{-1}}]$	0.05	0.2	0.1	0.19
S-TeH	k_{est}	$[\mathrm{Average\ individuals\ m^{-2}\ d^{-1}}]$	300	3000	365	2933
S-TeH	k_{root}	$[-]$	0.005	0.025	0.01	0.0097
S-TeH	$V_{max,up}$	$[\mathrm{gN\ gC^{-1}\ d^{-1}}]$	4	6	5.51	5.85
S-TeH	β_{root}	$[-]$	0.93	0.98	0.943	0.962
S-TeH	$C_{max,BNF}$	$[-]$	0.004	0.08	0.03	0.045
S-TeH	k_{store}	$[-]$	0.05	0.3	0.05	0.19
S-TeH	$N_{max,BNF}$	$[\mathrm{gN\ m^{-2}\ d^{-1}}]$	0.01	1	0.5	0.77
S-TeH	f_{avoid}	$[-]$	0.5	1	1	0.84
R-TeH	g_{min}	$[\mathrm{mm\ s^{-1}}]$	0.7	2	0.8	0.86
R-TeH	SLA	$[\mathrm{m^2\ gC^{-1}}]$	0.14	0.35	0.3	0.344874
R-TeH	$lmro$	$[-]$	0.3	1	0.8	0.33
R-TeH	LAI_{sapl}	$[-]$	0.005	0.03	0.01	0.016
R-TeH	E_{max}	$[\mathrm{mm\ d^{-1}}]$	6	12	7	8.3
R-TeH	k_{light}	$[-]$	0.2	0.5	0.4	0.28
R-TeH	$mort_{max}$	$[\mathrm{Average\ individuals\ m^{-2}\ d^{-1}}]$	0.05	1	0.2	0.81
R-TeH	k_{est}	$[\mathrm{Average\ individuals\ m^{-2}\ d^{-1}}]$	3000	6000	3650	5418
R-TeH	k_{root}	$[-]$	0.005	0.025	0.01	0.021
R-TeH	$V_{max,up}$	$[\mathrm{gN\ gC^{-1}\ d^{-1}}]$	4	6	5.51	5.25
R-TeH	β_{root}	$[-]$	0.9	0.95	0.943	0.919
R-TeH	$C_{max,BNF}$	$[-]$	0.004	0.08	0.03	0.022
R-TeH	k_{store}	$[-]$	0.05	0.3	0.05	0.16
R-TeH	$N_{max,BNF}$	$[\mathrm{gN\ m^{-2}\ d^{-1}}]$	0.01	1	0.5	0.38
R-TeH	f_{avoid}	$[-]$	0.5	1	1	0.68
C-PoH	g_{min}	$[\mathrm{mm\ s^{-1}}]$	0.7	2	0.8	1.16
C-PoH	SLA	$[\mathrm{m^2\ gC^{-1}}]$	0.04	0.14	0.1	0.75055
C-PoH	$lmro$	$[-]$	0.7	1.5	0.8	0.94
C-PoH	LAI_{sapl}	$[-]$	0.05	0.3	0.1	0.226
C-PoH	E_{max}	$[\mathrm{mm\ d^{-1}}]$	6	12	7	10.75
C-PoH	k_{light}	$[-]$	0.4	0.8	0.5	0.49
C-PoH	$mort_{max}$	$[\mathrm{Average\ individuals\ m^{-2}\ d^{-1}}]$	0.09	0.4	0.1	0.34
C-PoH	k_{est}	$[\mathrm{Average\ individuals\ m^{-2}\ d^{-1}}]$	300	3000	365	2047
C-PoH	k_{root}	$[-]$	0.005	0.025	0.01	0.013
C-PoH	$V_{max,up}$	$[\mathrm{gN\ gC^{-1}\ d^{-1}}]$	4	6	5.51	5.09
C-PoH	β_{root}	$[-]$	0.9	0.95	0.943	0.932
C-PoH	$C_{max,BNF}$	$[-]$	0.004	0.08	0.03	0.03
C-PoH	k_{store}	$[-]$	0.05	0.3	0.05	0.11
C-PoH	$N_{max,BNF}$	$[\mathrm{gN\ m^{-2}\ d^{-1}}]$	0.01	1	0.5	0.5
C-PoH	f_{avoid}	$[-]$	0.5	1.5	1	0.7

Table S2 (continued): PFT-specific minimum, maximum, default and optimised values for calibrated parameters for the competitive (C), stress tolerant (S) and ruderal (R) PFTs for tropical (Tr), temperate (Te) and polaar (Po) climates. All PFTs are herbaceous (H). All parameters are explained in 'Overview of LPJmL-CSR' for parameter definitions before this table.

PFT	Parameter	Unit	Min	Max	Def	Opt
S-PoH	g_{min}	$[\mathrm{mm\ s^{-1}}]$	0.3	1	0.8	0.63
S-PoH	SLA	$[\mathrm{m^2\ gC^{-1}}]$	0.01	0.04	0.03	0.589573
S-PoH	$lmro$	$[-]$	0.3	1	0.8	0.64
S-PoH	LAI_{sapl}	$[-]$	0.05	0.3	0.1	0.272
S-PoH	E_{max}	$[\mathrm{mm\ d^{-1}}]$	3.5	8	7	5.66
S-PoH	k_{light}	$[-]$	0.4	0.8	0.5	0.53
S-PoH	$mort_{max}$	$[\mathrm{Average\ individuals\ m^{-2}\ d^{-1}}]$	0.05	0.2	0.1	0.13
S-PoH	k_{est}	$[\mathrm{Average\ individuals\ m^{-2}\ d^{-1}}]$	300	3000	365	2576
S-PoH	k_{root}	$[-]$	0.005	0.025	0.01	0.011
S-PoH	$V_{max,up}$	$[\mathrm{gN\ gC^{-1}\ d^{-1}}]$	4	6	5.51	5.18
S-PoH	β_{root}	$[-]$	0.93	0.98	0.943	0.946
S-PoH	$C_{max,BNF}$	$[-]$	0.004	0.08	0.03	0.03
S-PoH	k_{store}	$[-]$	0.05	0.3	0.05	0.21
S-PoH	$N_{max,BNF}$	$[\mathrm{gN\ m^{-2}\ d^{-1}}]$	0.01	1	0.5	0.5
S-PoH	f_{avoid}	$[-]$	0.5	1.5	1	0.7
R-PoH	g_{min}	$[\mathrm{mm\ s^{-1}}]$	0.6	2	0.8	1.45
R-PoH	SLA	$[\mathrm{m^2\ gC^{-1}}]$	0.14	0.35	0.3	0.726572
R-PoH	$lmro$	$[-]$	0.3	1	0.8	0.74
R-PoH	LAI_{sapl}	$[-]$	0.05	0.1	0.08	0.075
R-PoH	E_{max}	$[\mathrm{mm\ d^{-1}}]$	6	12	7	9.02
R-PoH	k_{light}	$[-]$	0.2	0.5	0.4	0.45
R-PoH	$mort_{max}$	$[\mathrm{Average\ individuals\ m^{-2}\ d^{-1}}]$	0.05	1	0.2	0.4
R-PoH	k_{est}	$[\mathrm{Average\ individuals\ m^{-2}\ d^{-1}}]$	3000	6000	3650	4415
R-PoH	k_{root}	$[-]$	0.005	0.025	0.01	0.0137
R-PoH	$V_{max,up}$	$[\mathrm{gN\ gC^{-1}\ d^{-1}}]$	4	6	5.51	5.36
R-PoH	β_{root}	$[-]$	0.9	0.95	0.943	0.919
R-PoH	$C_{max,BNF}$	$[-]$	0.004	0.08	0.03	0.03
R-PoH	k_{store}	$[-]$	0.05	0.3	0.05	0.27
R-PoH	$N_{max,BNF}$	$[\mathrm{gN\ m^{-2}\ d^{-1}}]$	0.01	1	0.5	0.5
R-PoH	f_{avoid}	$[-]$	0.5	1.5	1	0.7

Livestock grazing. The livestock module is used to calculate dry matter intake of ruminants using lactating dairy cows as a generic representation of livestock (11). Dry matter intake depends on forage quality, defined by the leaf C:N ratio and livestock density. Based on forage quality, the model determines how C and N are distributed between respiration, feces, urine and milk (as a proxy for a product). For this, we used a global dataset prescribing spatially explicit but temporally constant livestock densities over the entire simulation period (14). The data were derived from estimates of grazing levels at regional scales and production system levels from (15) using a set of simulations presented in (11).

For this study, we combined the implementation of CSR strategies (8) with the livestock module (11), which required the introduction of a factor to account for grazing avoidance of the different strategies. This factor is used to adjust the carbon intake from each PFT so that the C-PFT, which follows a grazing tolerance rather than an avoidance strategy (16, 17), is grazed more heavily compared to the S- and R-PFTs that follow a grazing avoidance strategy (Eq. 1).

$$f_{grazed,t,p} = \frac{C_{in,t} \cdot f_{avoid,p} \cdot C_{leaf,t,p}}{\sum_{i=1}^{NPFT} C_{leaf,t,i} \cdot f_{avoid,i}} \; \forall \; p \; \in \; NPFT \qquad [1]$$

$f_{grazed,t,PFT}$ is the share of total carbon intake of a PFT at timestep t, C_{in} is the total carbon intake, $f_{avoid,PFT}$ is the PFT-specific unitless avoidance factor, $C_{leaf,PFT}$ is the PFT-specific leaf carbon in gC m^{-2} and $NPFT$ is the number of established PFTs. This ensures that the total carbon intake remains as derived by Eq. 27 from 11. However, due to different carbon-to-nitrogen ratios of the different PFTs, the total nitrogen intake may differ. As a consequence, the nitrogen allocated to feces, carbon and nitrogen allocated to milk and urine, and carbon respired are adjusted following Eq. 42, 43, 48, 50 and 53 in 11.

Evaluation of model performance. We evaluate model performance before (prior) and after calibration (best) against observations to show whether the calibration was successful.

Comparing the observations of GPP and foliage projective cover for all grid cells not used in the calibration to simulations using the default (prior) and the optimised (best) parameter values showed minor changes for GPP but a substantial reduction in the difference to observations for foliage projective cover of C-, S- and R-PFTs. For GPP, the median difference between observation and simulation was 0.16 kgC m^{-2} yr^{-1} for the prior and 0.18 kgC m^{-2} yr^{-1} for the best parameter set. Median values, the 25th and 75th percentiles, and the interquartile range were similar between the prior and the best (Fig. S1 a). However, the best parameter set strongly overestimated annual GPP in more cases than the prior parameter set, as indicated by the outlier values above the upper interquartile boundary. Monthly GPP was up to 0.4 kgC m^{-2} yr^{-1} lower or higher than observations (Fig. S1 b) for both the prior and best parameter sets. Similar to the annual GPP, overestimations of monthly GPP are more common using the best parameter set. However, the difference is small for a majority of data points, as shown by the density plot in Fig. S1 b. The main increase in GPP between the prior and the best parameter set occurs in the tropics (Fig. S3). For example, in the Congo basin, monthly GPP is up to 0.3 kgC m^{-2} yr^{-1} higher using the best parameters (see Fig. S3 a and c). However, observations here generally show quite low grassland GPP, potentially underestimating productivity. While we believe that the representation of GPP by the model can be improved, we are confident that the results using the best parameter set are sufficient to investigate the role of functional diversity for grassland ecosystem services.

Foliage projective cover of the C-, S- and R-PFTs differed strongly between the prior and best parameter sets. Overall, the median difference to the observations was lower for the best parameter set. It was -0.33, -0.31, and 0.63 m^2 m^{-2} for the prior and 0.18, 0.01 and -0.23 m^2 m^{-2} for the best parameter set for the C-, S- and R-PFTs, respectively (Fig. S1 c). Qualitatively, the foliage projective cover of the C-PFTs remains higher than the observations, while that of the R-PFTs is lower. The S-PFTs' foliage projective cover matches the observations best. However, for all three PFT types, the difference to observations shows a wide range. Comparison by latitude showed that the foliage projective cover of the R-PFTs was strongly overestimated at the expense of the C- and S-PFTs using the prior parameter set (Fig. S1 d). In contrast, using the best parameter set, the foliage projective cover of the C-PFTs is overestimated at the expense of the other PFTs. In the high latitudes of the southern hemisphere, overestimations were the largest and at the expense of both the S- and R-PFTs. At lower latitudes, the foliage projective cover of the S-PFTs matches observations well, while that of the C-PFTs is overestimated at the expense of the R-PFT. In the high latitudes of the northern hemisphere, differences are smaller and the foliage projective covers mainly agree well with observations. An exception is the latitudes between 60 and 66°N where the share of managed grasslands is negligible except on Iceland, which shows a strong overestimation of the foliage projective cover of C-PFTs (Fig. S4 j).

Using the prior parameter set, the R-PFT was dominant across different climate zones except in India. The best parameter set results in regionally different foliage projective cover distributions (Fig. S4). Using the best parameter set, the foliage projective cover of C-PFTs is close to 1 in Europe, south-eastern USA, Uruguay, southern Brazil, eastern Argentina as well as parts of South Africa, Australia and the Himalayas. These areas also correspond to where LPJmL-CSR overestimates most overestimates the foliage projective cover of the C-PFTs relative to observations. Towards the tropics as well as the higher latitudes of the northern hemisphere, the share of C-PFTs decreases. Here, both the R- and S-PFTs have a substantial share of foliage projective cover with values up to approximately 0.25 and 0.5, respectively. However, the model still underestimates the R-PFTs relative to observations while overestimating the S-PFTs. In the subtropics and tropics, the R-PFTs are barely present and the S-PFTs have a foliage projective cover of up to approximately 0.75, resulting in overestimation of both S- and C-PFTs and underestimation of the R-PFTs.

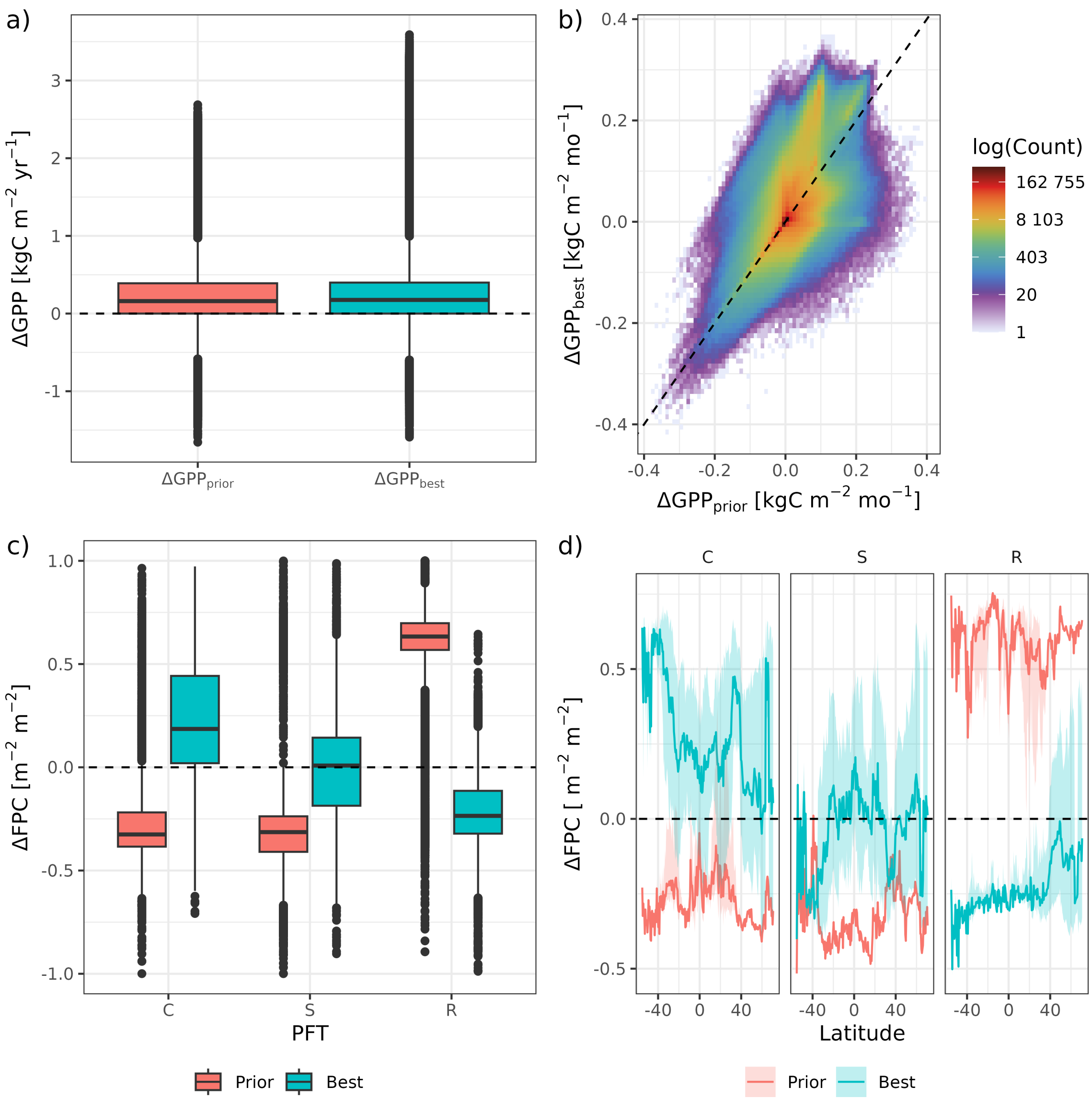

Fig. S1. Boxplots (a,c), density plot (b) and latitudinal averages (d) of the difference between simulated and observed annual (a) and monthly (b) GPP between 2000 and 2016 and annual foliage projective cover (c,d) data for the parameter set before (prior, blue) and after (best, red) the calibration. For the latitude plot, lines show the differences to the 2000 to 2016 average of simulations results and shaded areas show the 5th to 95th percentile.

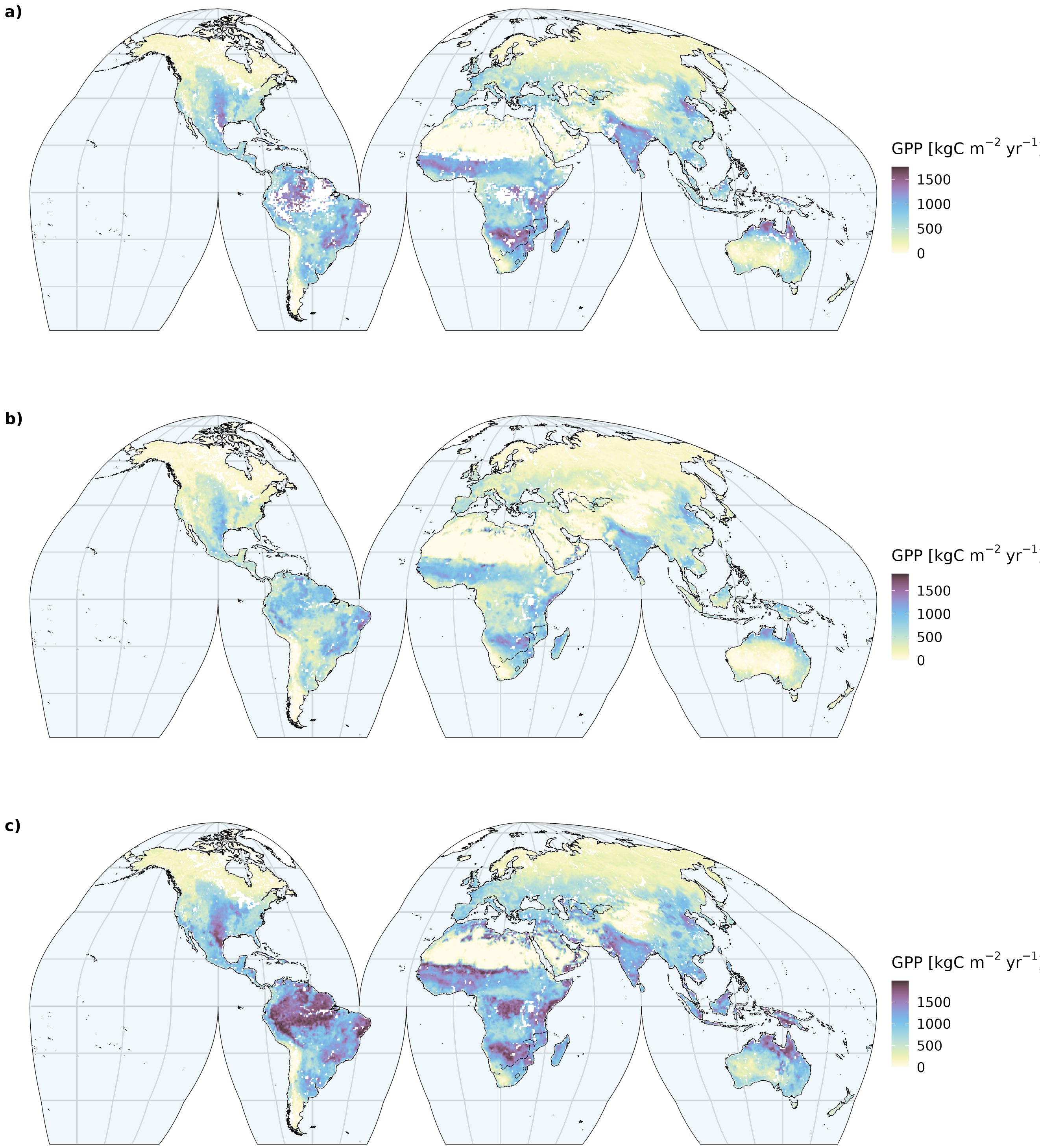

Fig. S2. Time-series average (a), 5th (b) and 95th (c) percentile of the annual herbaceous GPP calculated from (18).

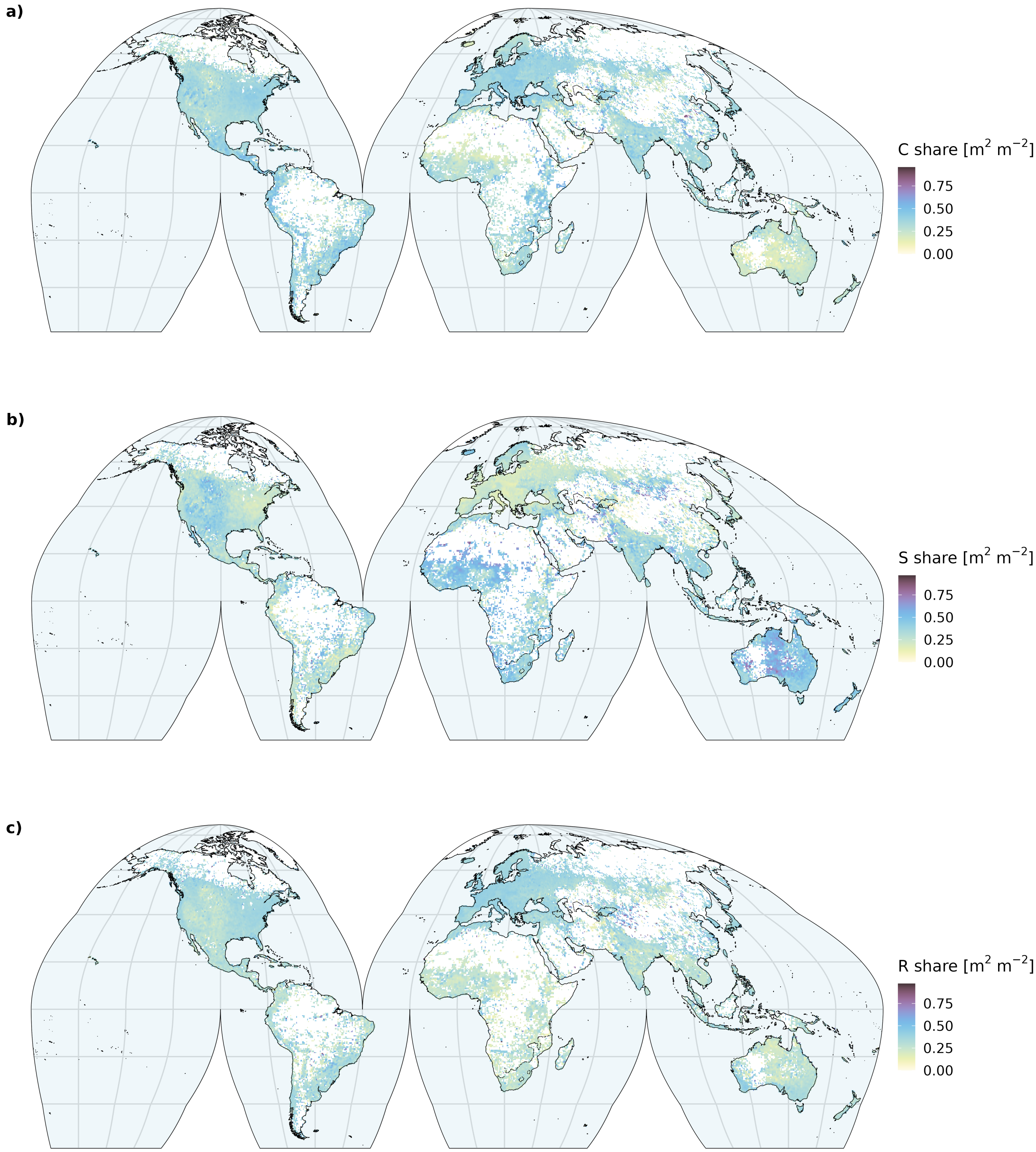

Fig. S3. Community share of the C- (a), S- (b) and R-PFT (c) calculated using the StrateFy approach and the (19) data.

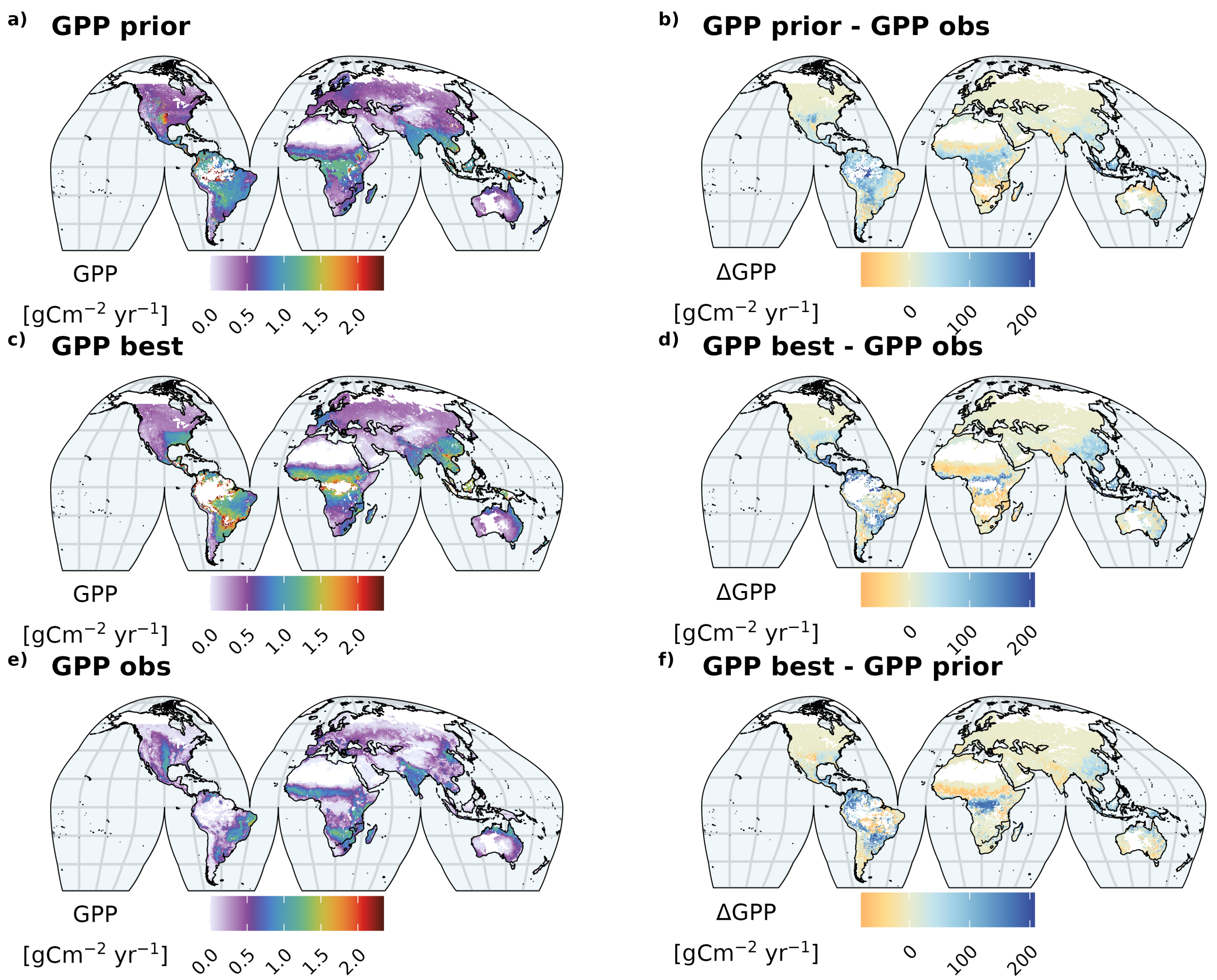

Fig. S4. 2000 to 2016 average GPP for the parameter sets before (a) and after (c) calibration and observations (e) as well as difference between the parameters before the calibration and observations (b), the parameter set after the calibration and observations (d) and the two parameter sets (f).

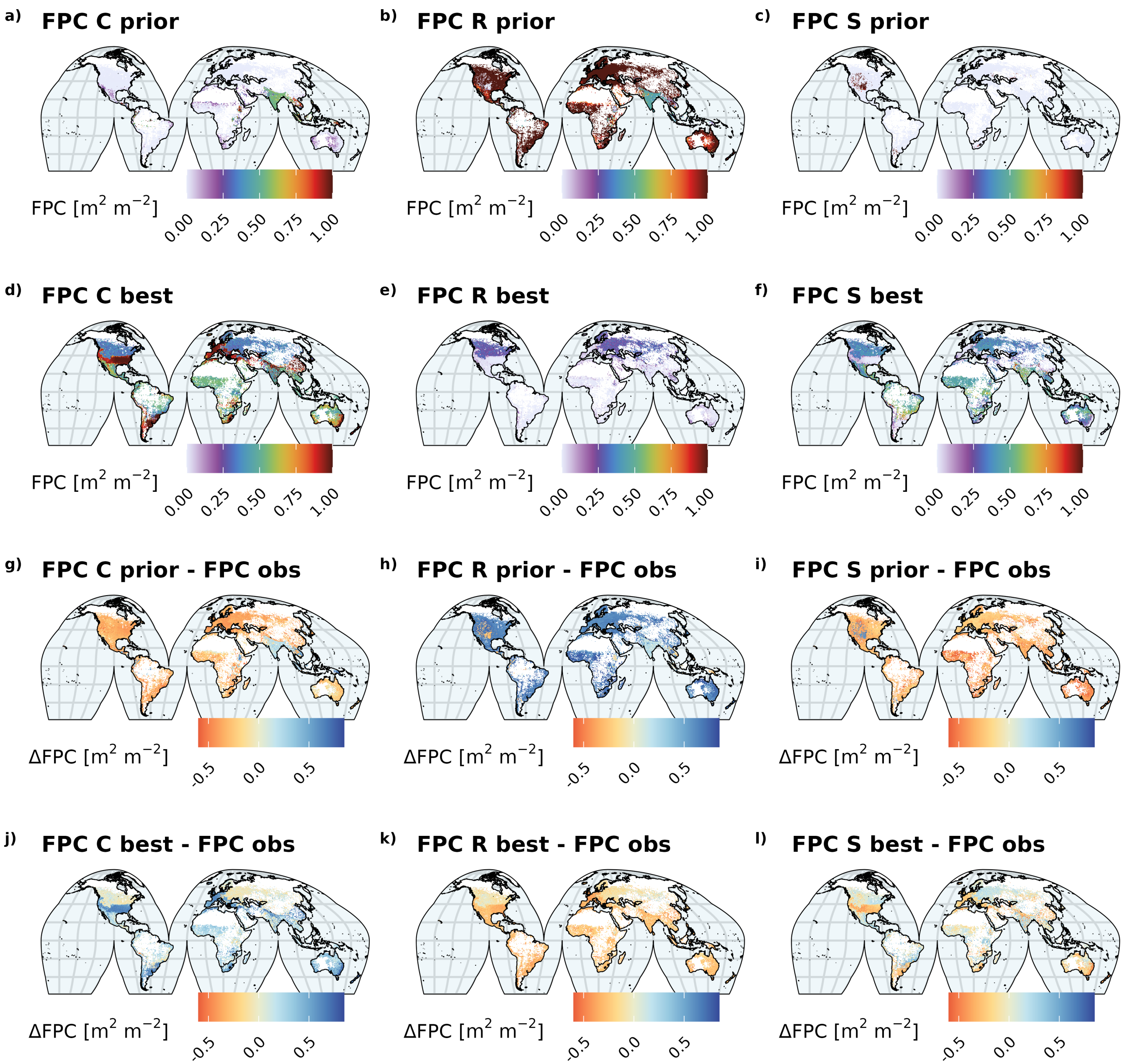

Fig. S5. foliage projective cover for each strategy for the parameter sets before (a, b, c) and after (d,e,f) calibration and difference to observations for the parameters before the calibration (g,h,i) and the parameter set after the calibration (j,k,l).

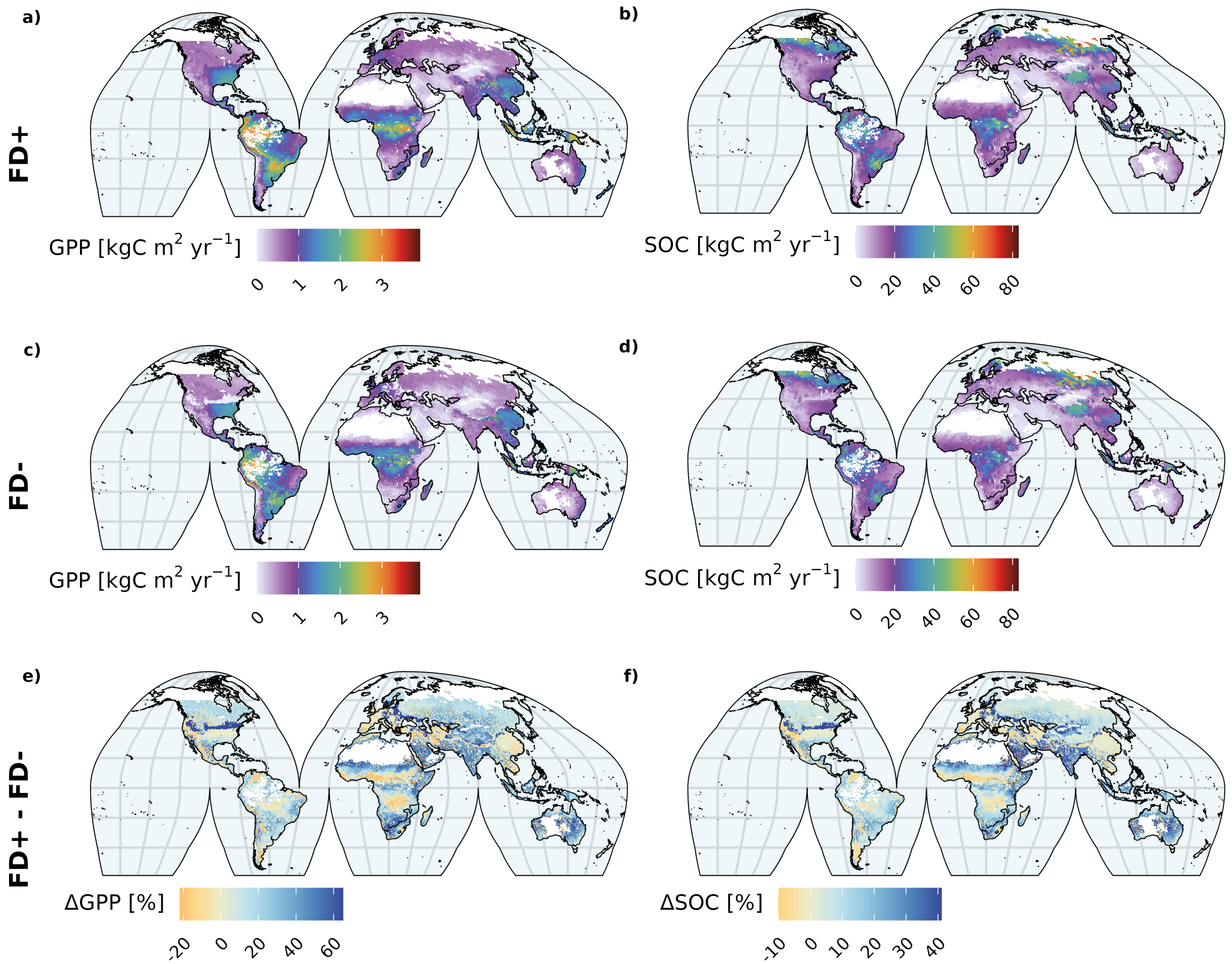

Fig. S6. Global distribution of GPP (a,c,e) and SOC (b,d,f) dependent on functional diversity for SSP3-7.0. Left panels show the average GPP for the scenarios (a) FD+, (b) FD- and (c) their difference, i.e. the net effect of a higher functional diversity. Right panels (b,d,f) show the respective results for SOC. All distributions show the 2091 to 2100 average of the respective output variable simulated using climate data from the MRI-ESM2-0.

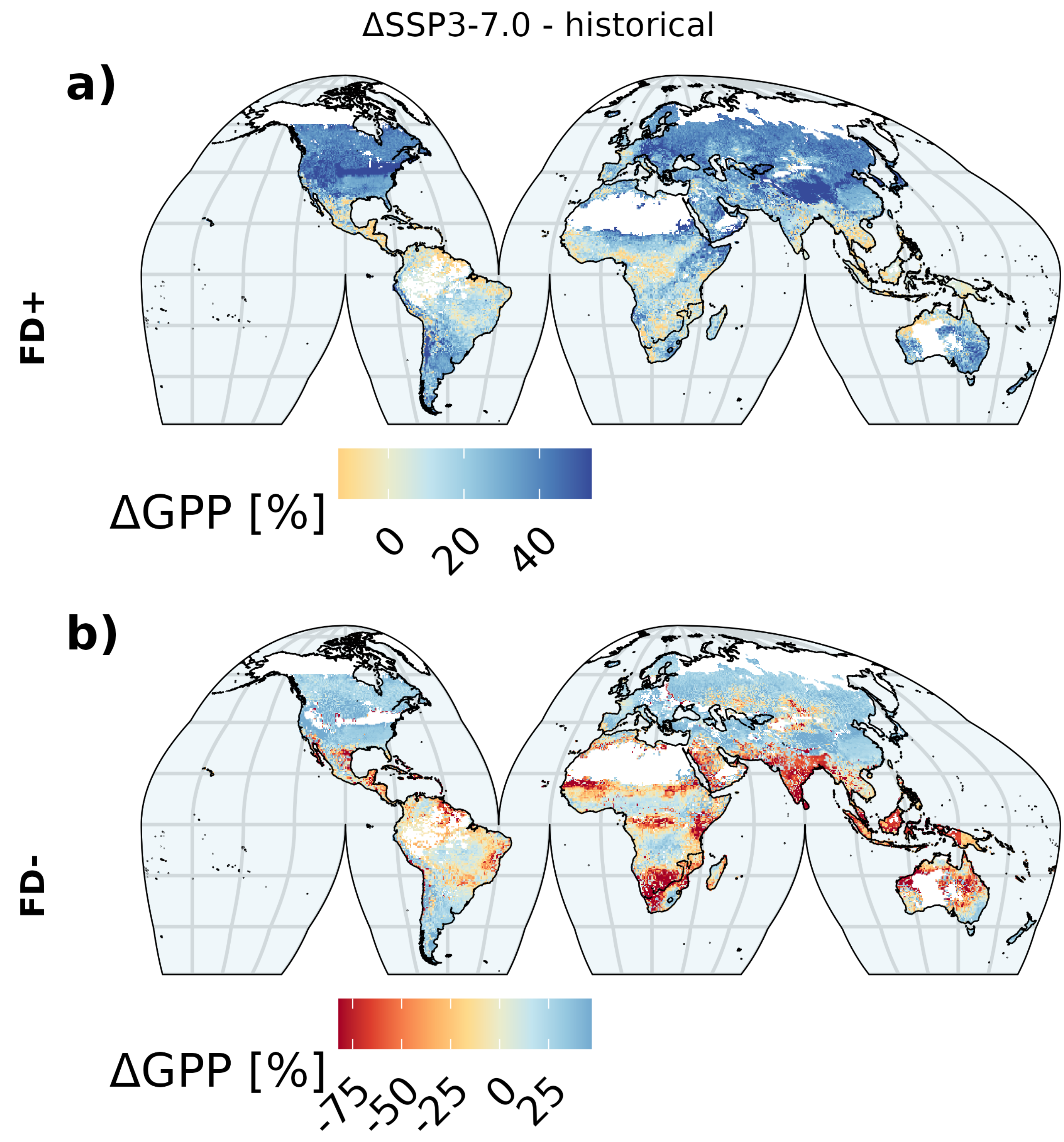

Fig. S7. Global distribution of the relative difference of GPP (a) and SOC (b) between the end of the historical time period (2005-2014) and the current century (2091-2100). All distributions show the time frame average of the respective output variable simulated using climate data from the MRI-ESM2-0.

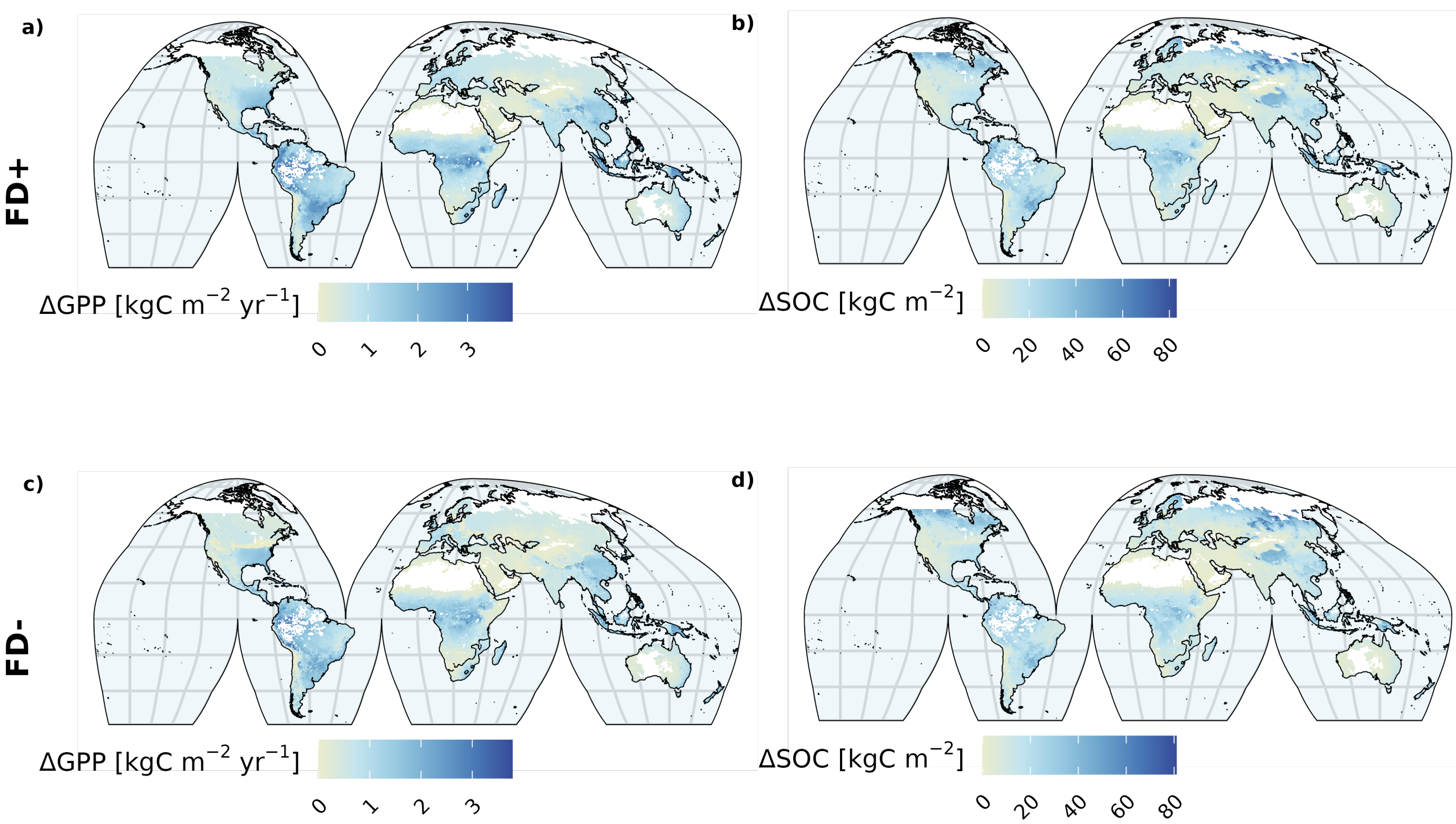

Fig. S8. Differences in GPP and SOC between SSP3-7.0 and SSP1-2.6 for the 2091-2100 average. Maps show SSP3-7.0 minus SSP1-2.6 for (a,b) GPP and SOC under FD+ and (c,d) GPP and SOC under FD-. All distributions show the time frame average of the respective output variable simulated using climate data from the MRI-ESM2-0.

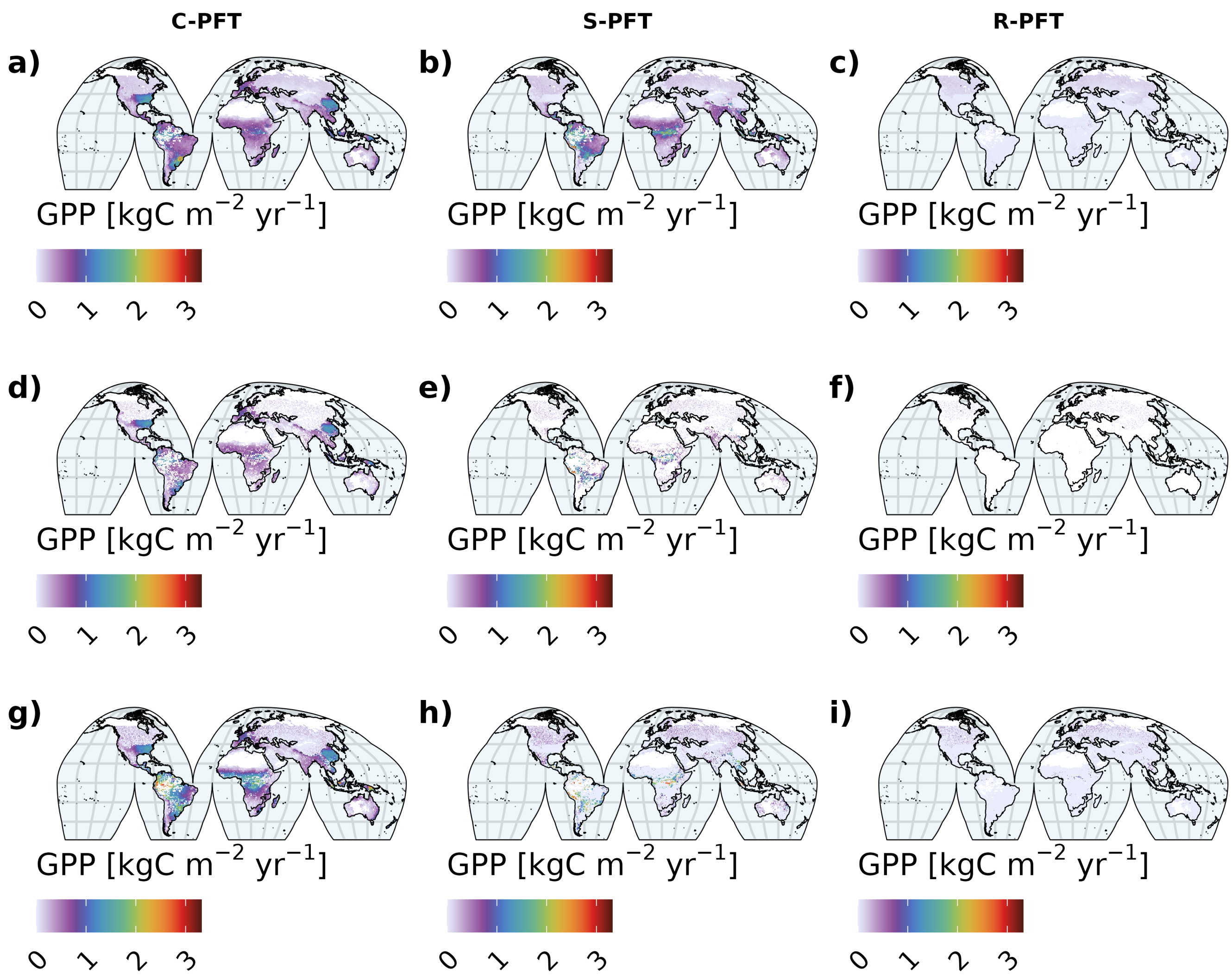

Fig. S9. Global distribution of PFT-specific GPP dependent on functional diversity. From left to right, panels show the distribution for the C- (a,d,g), S- (b,e,h) and R-PFTs (c,f,i). The first (a,b,c) and third (g,h,i) rows show the distribution using all PFTs present in FD+. The second row (d,e,f) shows the distribution using only the dominant PFT in FD+. The third row (g,h,i) shows the distribution for FD-. All distribution show the 2091 to 2100 average of the respective output variable simulated using climate data from the MRI-ESM2-0.

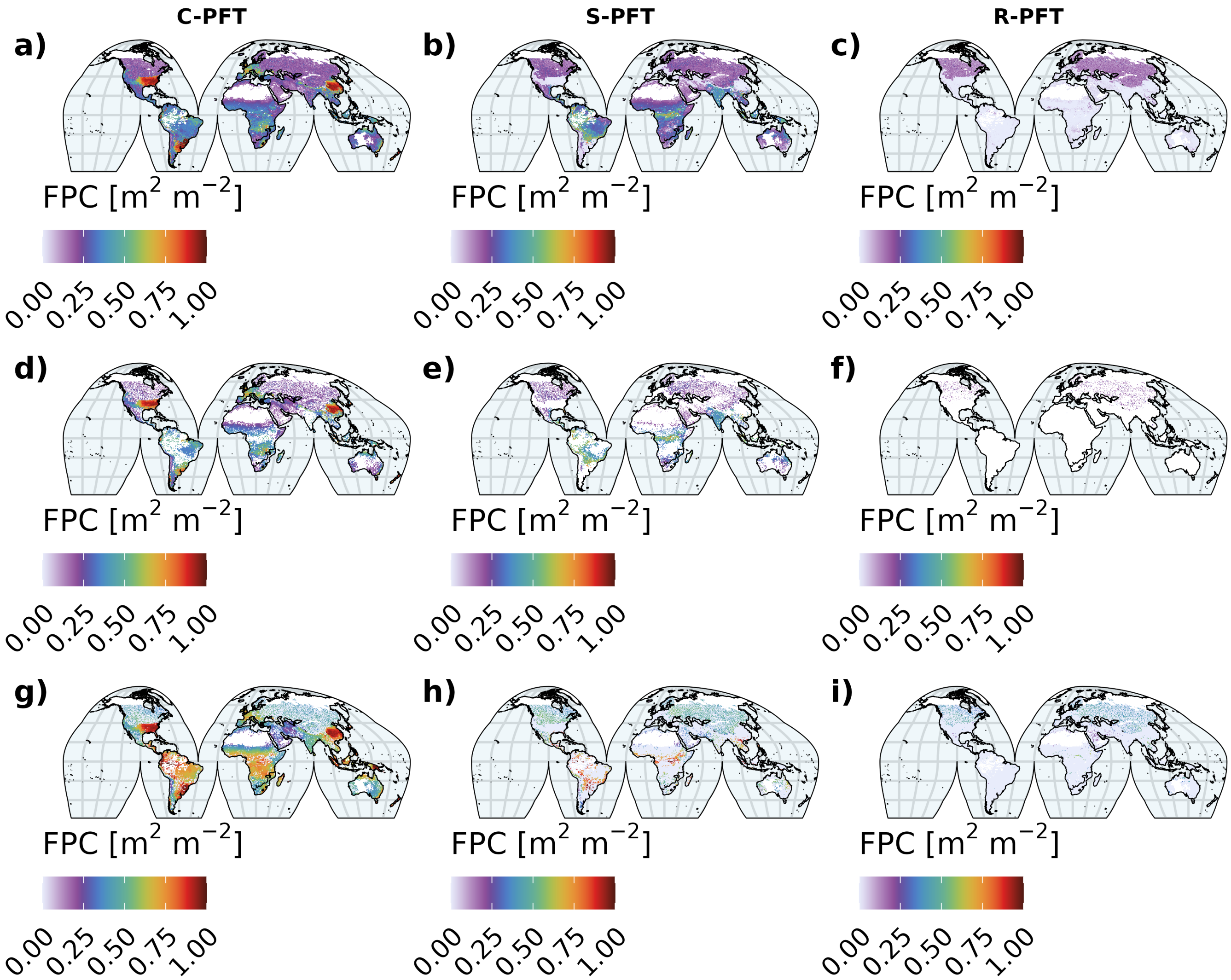

Fig. S10. Global distribution of PFT-specific foliage projective cover dependent on functional diversity. From left to right, panels show the distribution for the C- (a,d,g), S- (b,e,h) and R-PFTs (c,f,i). The first (a,b,c) and third (g,h,i) rows show the distribution using all PFTs present in FD+. The second row (d,e,f) shows the distribution using only the dominant PFT in FD+. The third row (g,h,i) shows the distribution for FD-. All distribution show the 2091 to 2100 average of the respective output variable simulated using climate data from the MRI-ESM2-0.

Representative example regions

We selected three representative example regions that cover a range of different climates and grassland types (see Tab. SI S3). The three example regions span distinct climate zones across the global grassland biome. Additionally, these regions exhibit spatial patterns that illustrate the simulated influence of functional diversity on managed grassland's response to climate change. These values represent 2091-2100 averages under SSP1-2.6. Uganda and South Sudan represent tropical grasslands with warm year-round temperatures (22-29°C) and moderate precipitation (600-1500 mm yr^{-1}). Germany represents temperate Central European grasslands with cooler temperatures (8-10°C) and moderate precipitation (600-1300 mm yr^{-1}). The Argentinian Pampas experience humid subtropical conditions with warm temperatures (14-21°C) and intermediate precipitation (400-1300 mm yr^{-1}). Under SSP3-7.0, all regions show warming of 1.5-3°C relative to SSP1-2.6, with the strongest increases in temperate zones (2-3°C) and more moderate warming in the tropics (1.5-2.5°C) (20). Precipitation projections show greater uncertainty, particularly in tropical regions where model agreement is lower (20). These contrasting climate trajectories enable assessment of how functional diversity influences grassland productivity and carbon storage under varying degrees of climate change.

Table S3. Mean temperature and annual precipitation for example regions (2091-2100) across SSP scenarios. Values represent ensemble mean across all general circulation models with standard error.

Region	Scenario	Temperature [℃]	Precipitation [mm yr^{-1}]
Uganda and South Sudan, Africa	SSP1-2.6	27.5 ± 0.1	1098 ± 14
	SSP3-7.0	30.1 ± 0.1	1289 ± 15
Germany, Europe	SSP1-2.6	10.5 ± 0.1	907 ± 16
	SSP3-7.0	13.5 ± 0.1	909 ± 14
Argentinian Pampas	SSP1-2.6	17.6 ± 0.1	937 ± 14
	SSP3-7.0	19.8 ± 0.1	1021 ± 14

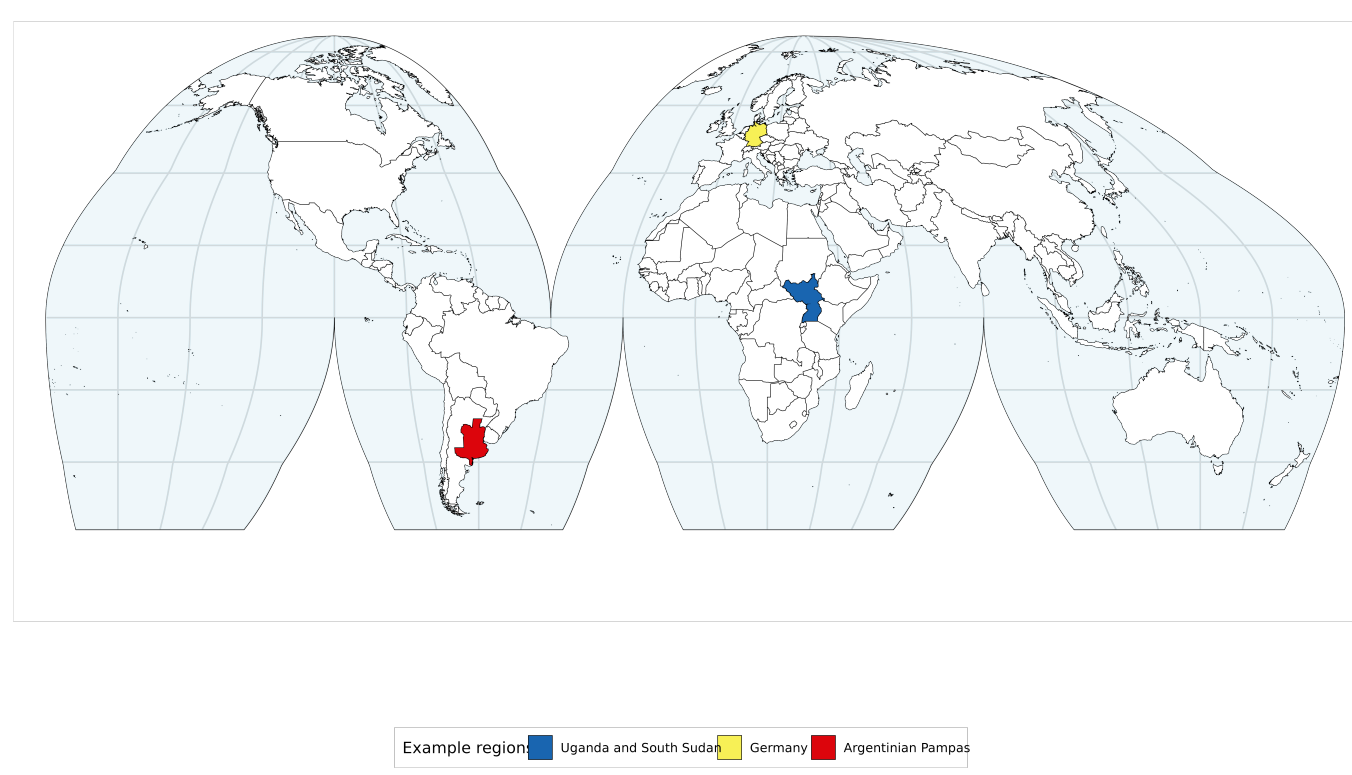

Fig. S11. Locations of the three example regions Uganda and South Sudan (blue), Germany (yellow) and the Argentinian Pampas (red).

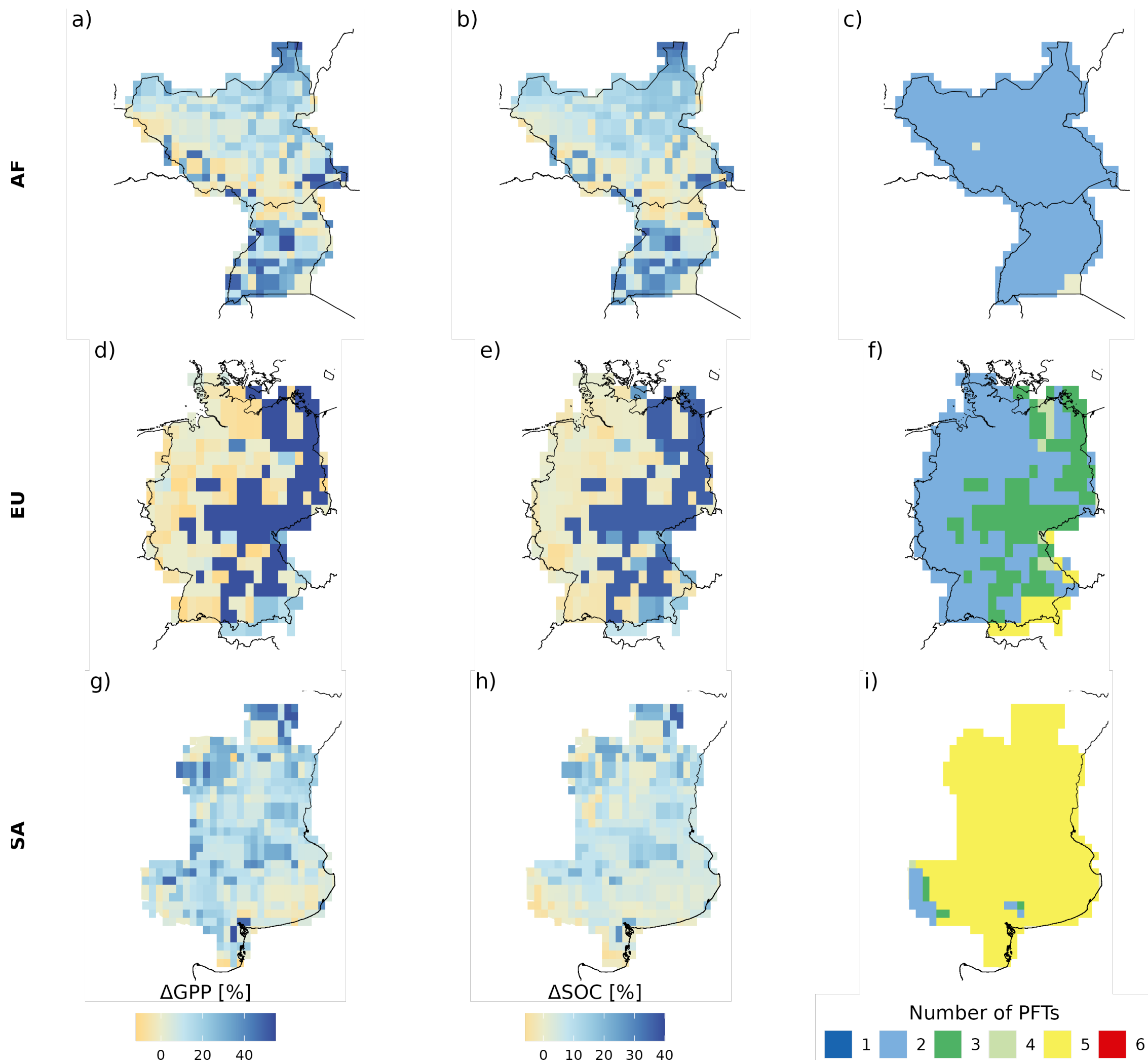

Fig. S12. Distribution of the relative change between FD+ and FD- for gross primary production (GPP, a,d,g) and soil organic carbon (SOC, b,e,h) and the absolute change of the number of PFT present (c,f,i) dependent on functional diversity for the three example regions (Uganda and South Sudan, Germany and the Argentinian Pampas). From top to bottom, the rows show the data for Uganda and South Sudan (a,b,c), Germany (d,e,f) and the Argentinian Pampas (g,h,i). From left to right, columns show the relative change in GPP (a,d,g) and SOC (b,e,h) and the absolute change in the number of PFTs (c,f,i). All distribution are based on the 2091 to 2100 average of the respective output variable simulated using climate data from the MRI-ESM2-0.

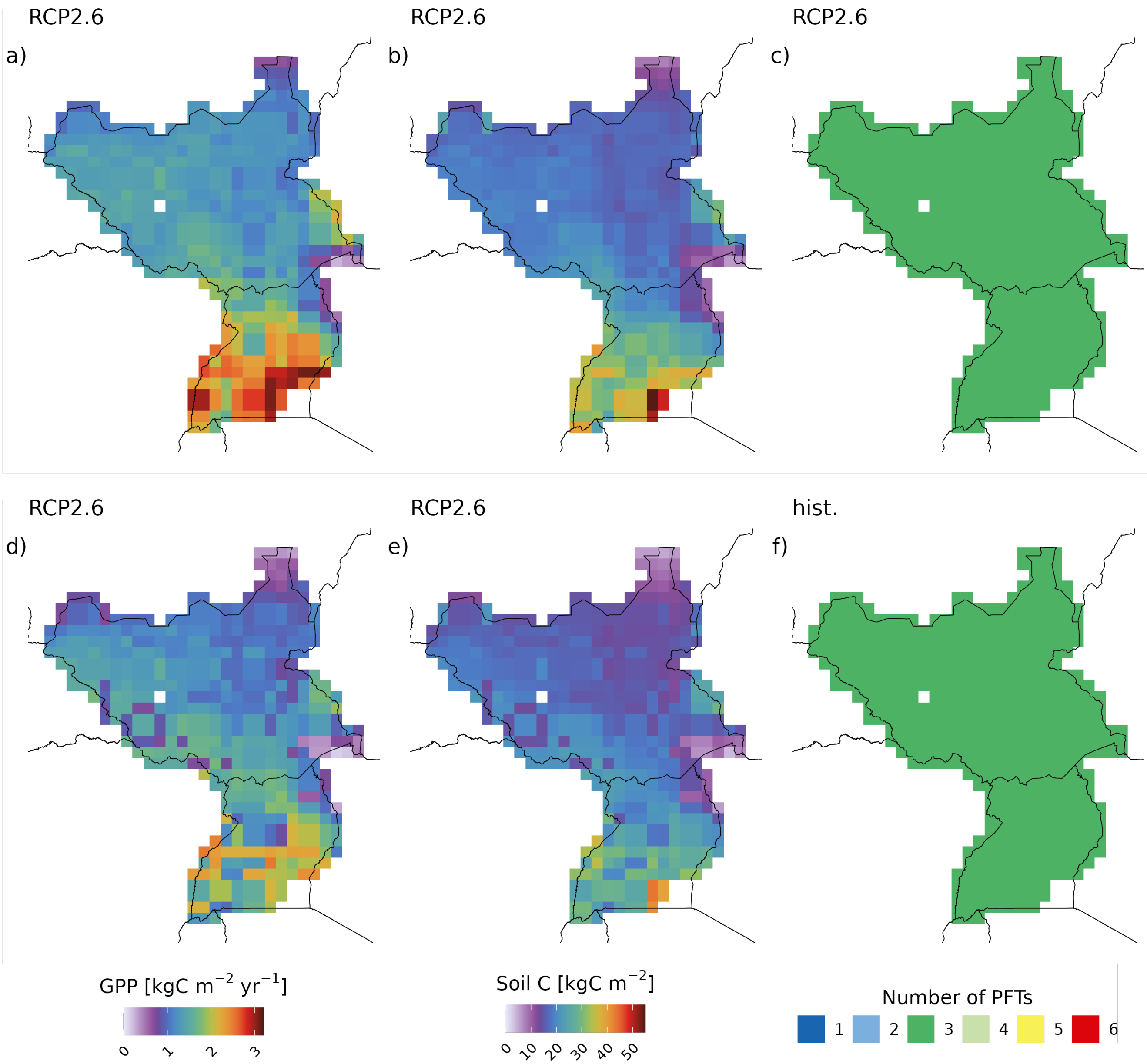

Fig. S13. Distribution of the absolute GPP (a,d), SOC (b,e) and the number of PFT present (c,f) dependent on functional diversity for Uganda and South Sudan. Panels show the data FD+ (a,b,c,f) and FD-(d,e). All distribution are based on the 2091 to 2100 average of the respective output variable simulated using climate data from the MRI-ESM2-0.

Uganda and South Sudan, Africa. Across the entire region, only the tropical PFTs were established and the number of PFTs is reduced by two in FD- compared to FD+, where three PFTs were present (Fig. S12 c). However, this does not translate into a homogeneous reduction of GPP and SOC (Fig. S12 a,b). Generally, GPP is higher in Uganda than in South Sudan (Fig. S13). This pattern is reflected in the relative changes in GPP from FD+ to FD-. In Uganda and northern and eastern South Sudan, GPP is up to 50% higher in FD+ compared to FD-, while it is up to 10% lower in the south-western part of South Sudan (Fig. S12 a). SOC storage shows a similar pattern, but values are only up to 30% higher and less than 10% lower in FD+ (Fig. S12 b). This pattern results from the different plant community compositions. In Uganda, the foliage projective cover of the subordinate C-PFT is still substantial and its removal cannot be compensated for by increased productivity of the dominant S-PFT. The pattern in South Sudan can be explained by the varying strength of the C-PFT dominance (Fig. S14 a and b). The foliage projective cover of the C-PFT is higher in the south-west than in the north-east (Fig. S14 a). A higher foliage projective cover of the C-PFT in FD+ means that its dominance is stronger. When this was the case, it was able to utilise resources better in both FD+ and FD-, leading to a higher GPP and SOC in FD- (Fig. S12 a and b). Here, the

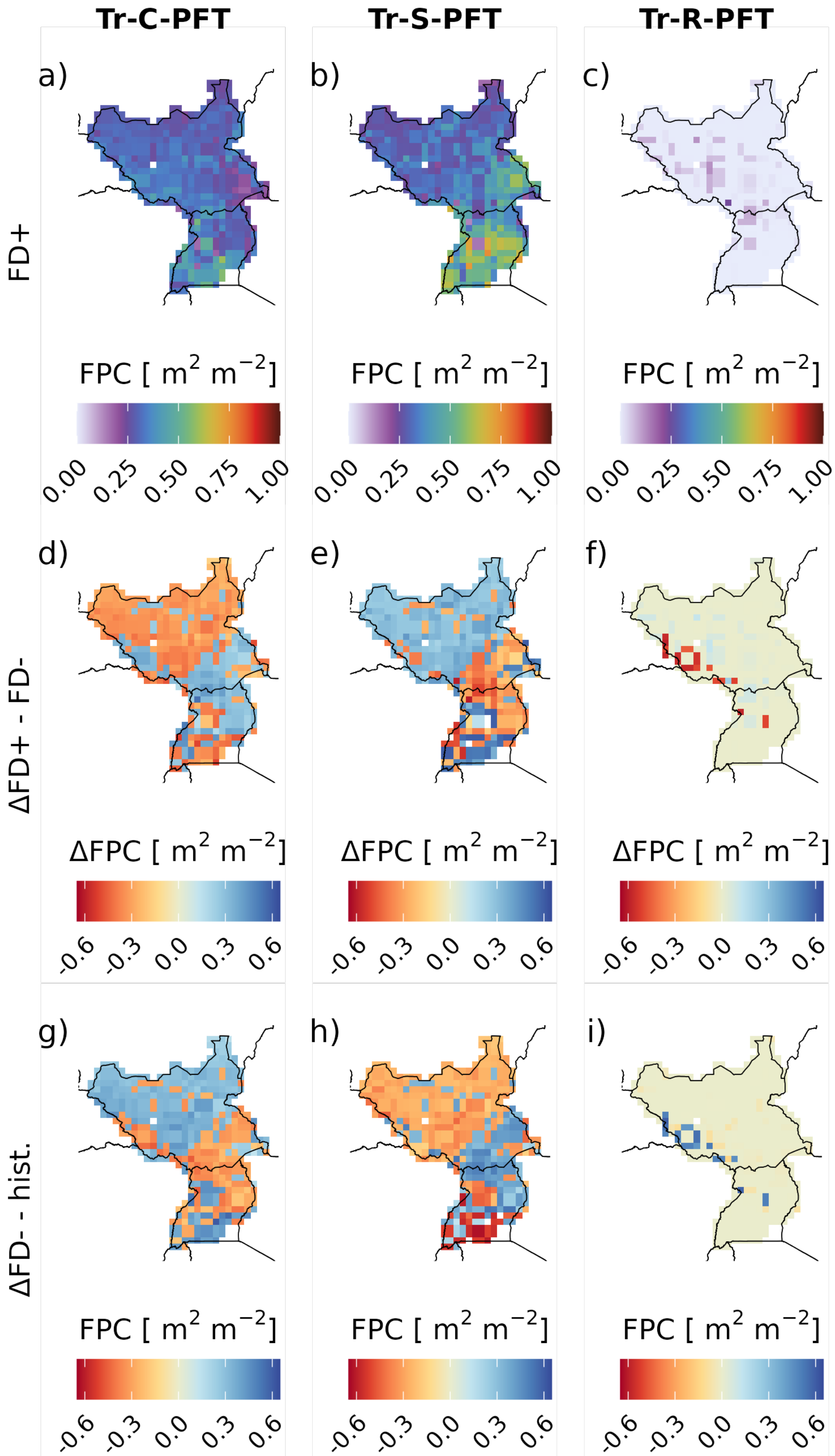

Fig. S14. Distribution of the absolute foliage projective cover (a,b,c) and relative difference between FD+ and FD- (c,d,e) and end of the historical time period (g,h,i) for the tropical C- (a,d,g), S- (b,e,h) and R-PFT (c,f,i) for Uganda and South Sudan. All distribution are based on the 2005 to 2014 and 2091 to 2100 average of the respective output variable simulated using climate data from the MRI-ESM2-0.

C-PFT benefits from reduced competition because the subordinate PFTs were removed. In the areas where the C-PFT is less dominant in the north-east, this advantage from reduced competition is weaker, and having the S-PFT as part of the community in FD+ is advantageous, resulting in higher GPP and SOC in FD+.

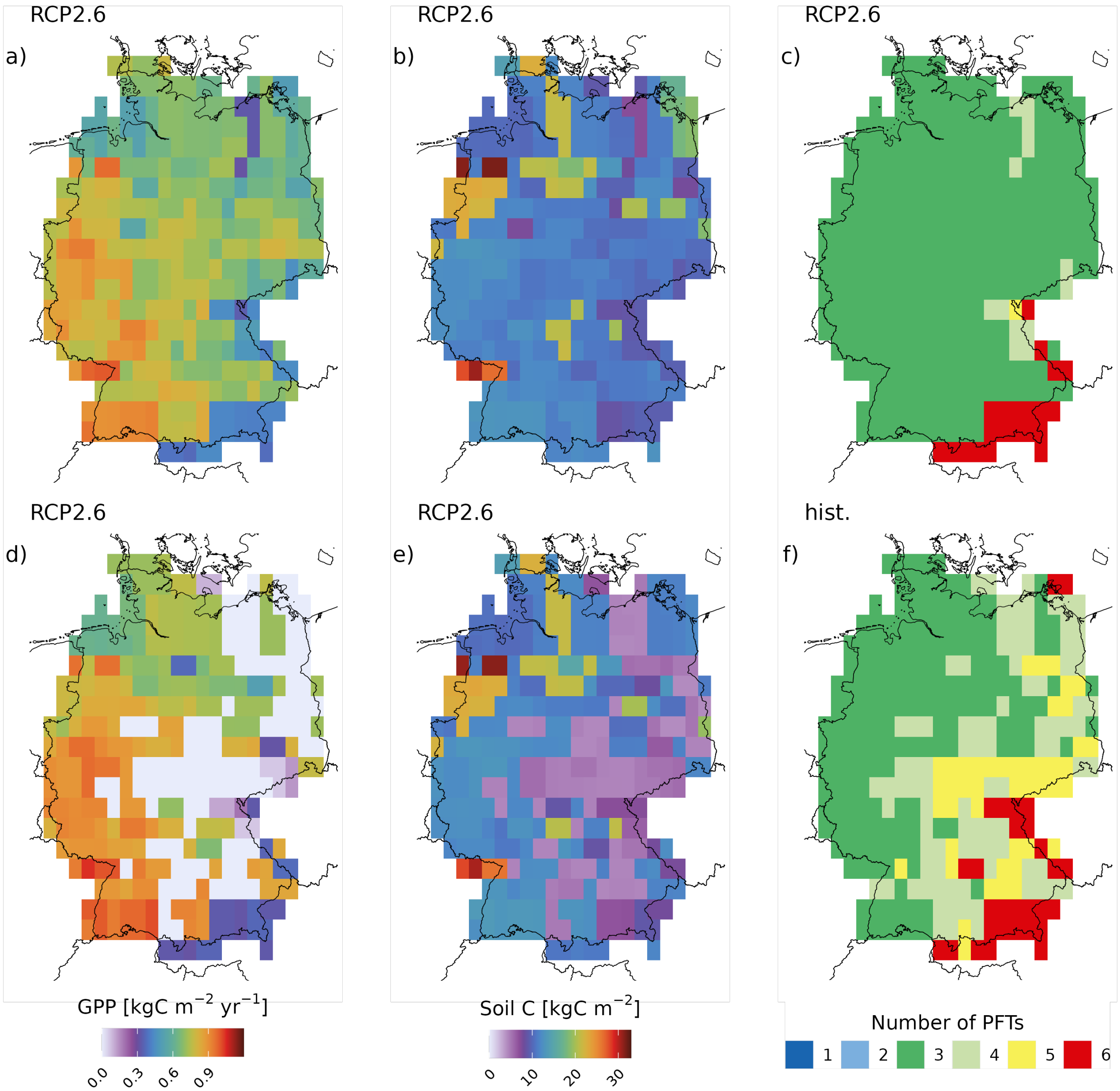

Fig. S15. Distribution of the absolute GPP (a,d), SOC (b,e) and the number of PFT present (c,f) dependent on functional diversity for Germany. Panels show the data FD+ (a,b,c,f) and FD-(d,e). All distribution are based on the 2091 to 2100 average of the respective output variable simulated using climate data from the MRI-ESM2-0.

Germany, Europe. In the second example region, Germany, absolute GPP is lower than in Uganda and South Sudan (compare Fig. S13 a and d with S15 a and d). However, the relative changes are similar, with up to 50% higher and 10% lower GPP values in FD+ and up to 30% higher and less than 10% lower SOC values (Fig. S12 d and e). The spatial patterns show higher GPP and SOC values in the east and south-east of Germany and lower values in the remaining areas. The spatial patterns of changes in GPP and SOC correspond to those of the number of PFTs lost (Fig. S12 f). Three PFTs are lost in the areas where GPP and SOC are higher in FD+ and two PFTs in those where FD+ values are lower. The strong loss of productivity in FD- in the south-east is caused by a complete loss of productivity of the historically dominant PFT (GPP of zero in south-east

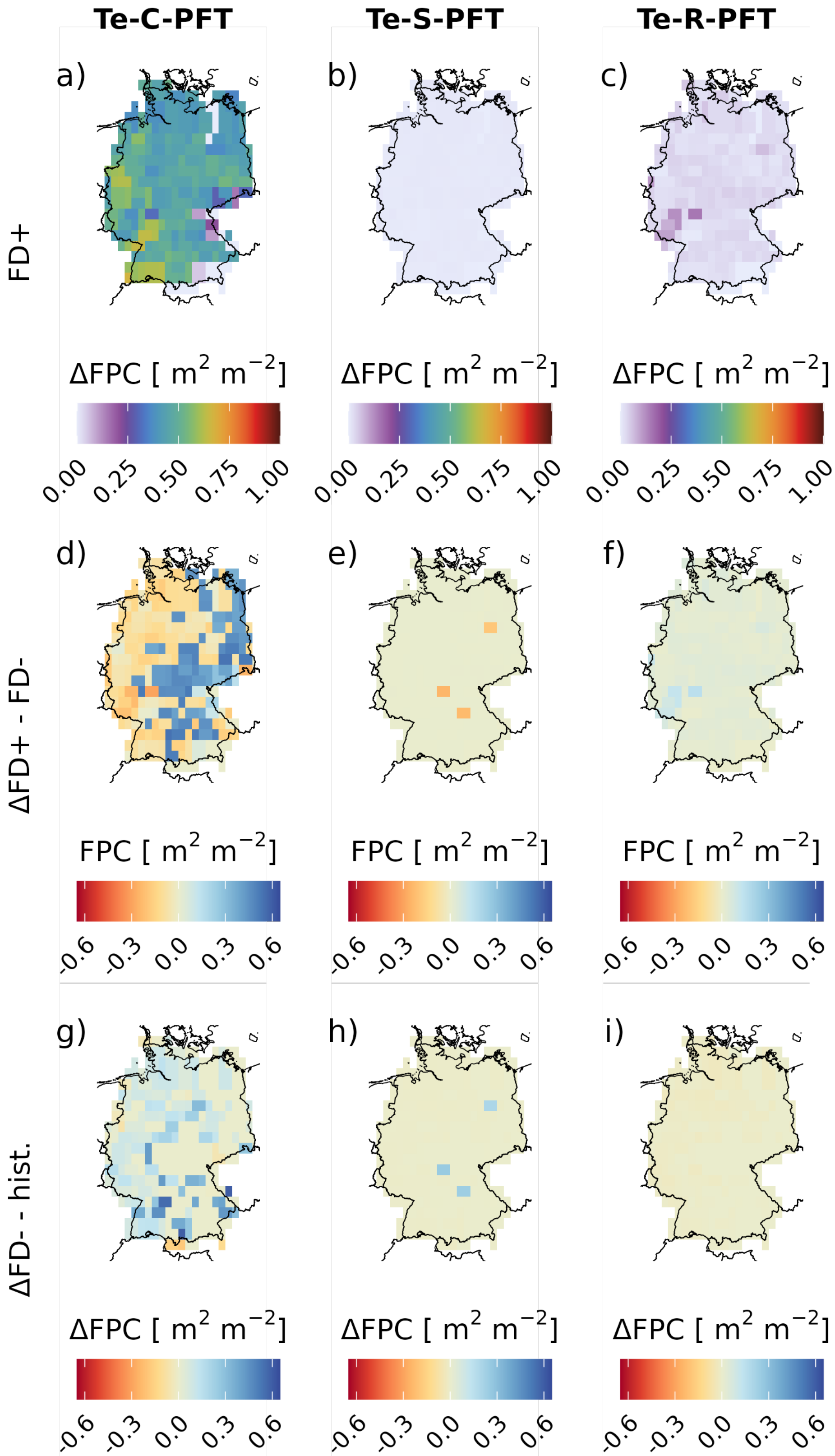

Fig. S16. Distribution of the absolute foliage projective cover (a,b,c) and relative difference between FD+ and FD- (c,d,e) and end of the historical time period (g,h,i) for the temperate C- (a,d,g), S- (b,e,h) and R-PFT (c,f,i) for Germany. All distribution are based on the 2005 to 2014 and 2091 to 2100 average of the respective output variable simulated using climate data from the MRI-ESM2-0.

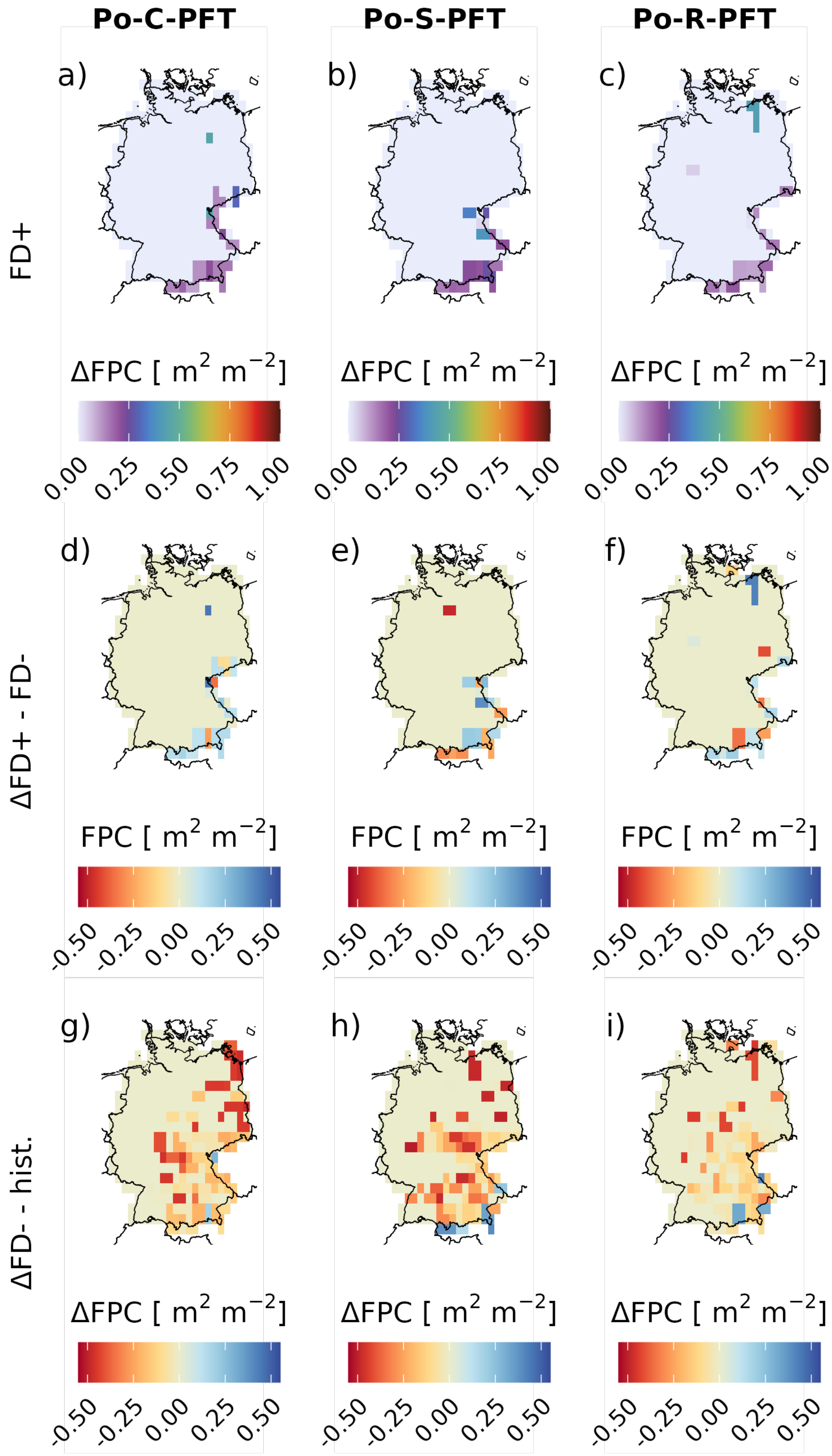

Fig. S17. Distribution of the absolute foliage projective cover (a,b,c) and relative difference between FD+ and FD- (c,d,e) and end of the historical time period (g,h,i) for the polar C- (a,d,g), S- (b,e,h) and R-PFT (c,f,i) for Germany. All distribution are based on the 2005 to 2014 and 2091 to 2100 average of the respective output variable simulated using climate data from the MRI-ESM2-0.

Germany in FD-; see Fig. S15 d). This points towards a different mechanism than in Uganda and South Sudan, where the dominant PFT was generally more productive in FD- but was still less productive compared to the mixture in FD+ in large areas. The community composition changes and PFTs are lost already in FD+ compared to the historical period from which the dominant PFT was determined (Fig. S15 c and f). This is caused by increasing temperatures, which render the polar PFTs that were present in the past unsuitable in the future. Due to overlapping bioclimatic limits that control the areas in which PFTs can establish, the polar C-PFT was dominant at the end of the historical simulation period in the south-east of Germany. However, under climate change, the temperatures in south-east Germany increase and exceed the bioclimatic limits of the polar PFTs, resulting in their removal from the community and complete loss of productivity compared to the historical period (Fig. S15 and S17). Generally, we think that the polar PFTs should not have been dominant in this region historically, which suggests the need for an adaptation of the bioclimatic limits. However, this nicely shows how climate change may lead to a loss of productivity if unsuitable PFTs cannot be replaced, leading to the breakdown of GPP. In the west and north of Germany, the temperate C-PFT is dominant (Fig. S16) and benefits from the removal of subordinate PFTs, allowing it to compensate for the loss of subordinate PFTs in FD-.

Argentinian Pampas, South America. Here, GPP increases from the south-east to the north-west and the values are similar to Uganda and South Sudan (compare Fig. S13 a and d, S15 a and d and S18 a and d). Generally, GPP and SOC are higher or similar in FD+, with values of up to 40% higher GPP and SOC in FD+ in extreme cases in the north (Fig. S12 g and h). In the south-eastern part of Buenos Aires, differences between FD+ and FD- are negligible. While the temperate C-PFT was strongly dominant across the example region except in the far north during the historical time period, in FD+ the tropical PFTs gain an advantage in larger parts of the region under climate change, which is beneficial for productivity (Fig. S19 a and d). While at the end of the century both the tropical and temperate PFTs can establish across almost the entire region, this was not the case during the historic time period (Fig. S18 c and f) In contrast to Germany, the higher productivity in FD+ is explained by shifts of the C3 and C4 PFT shares under climate change instead of a breakdown of the community. Historically, the temperate C-PFT is dominant in the majority of the example region and is therefore selected for FD-. Under climate change, the tropical C-PFT gains an advantage and becomes more important for the communities in large parts of the region in FD+, stabilising the community productivity (Fig. S19 d). In FD-, the historically dominant PFTs benefits from decreased resource competition (Fig. S20 and S19 g) but cannot compensate for the productivity gains of the mixture in large parts of the Argentinian Pampas.

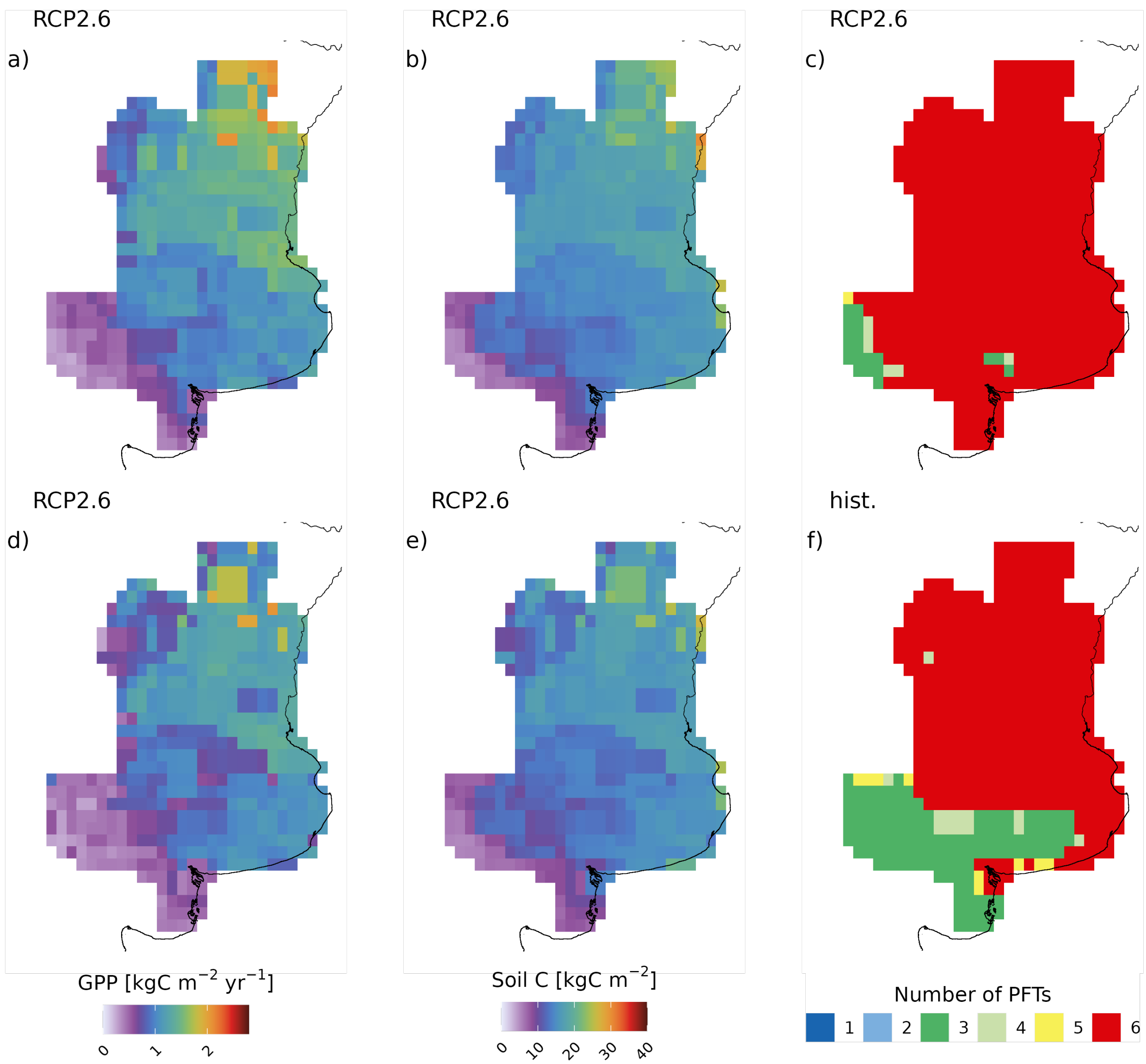

Fig. S18. Distribution of the absolute GPP (a,d), SOC (b,e) and the number of PFT present (c,f) dependent on functional diversity for the Argentinian Pampas. Panels show the data FD+ (a,b,c,f) and FD-(d,e). All distribution are based on the 2091 to 2100 average of the respective output variable simulated using climate data from the MRI-ESM2-0.

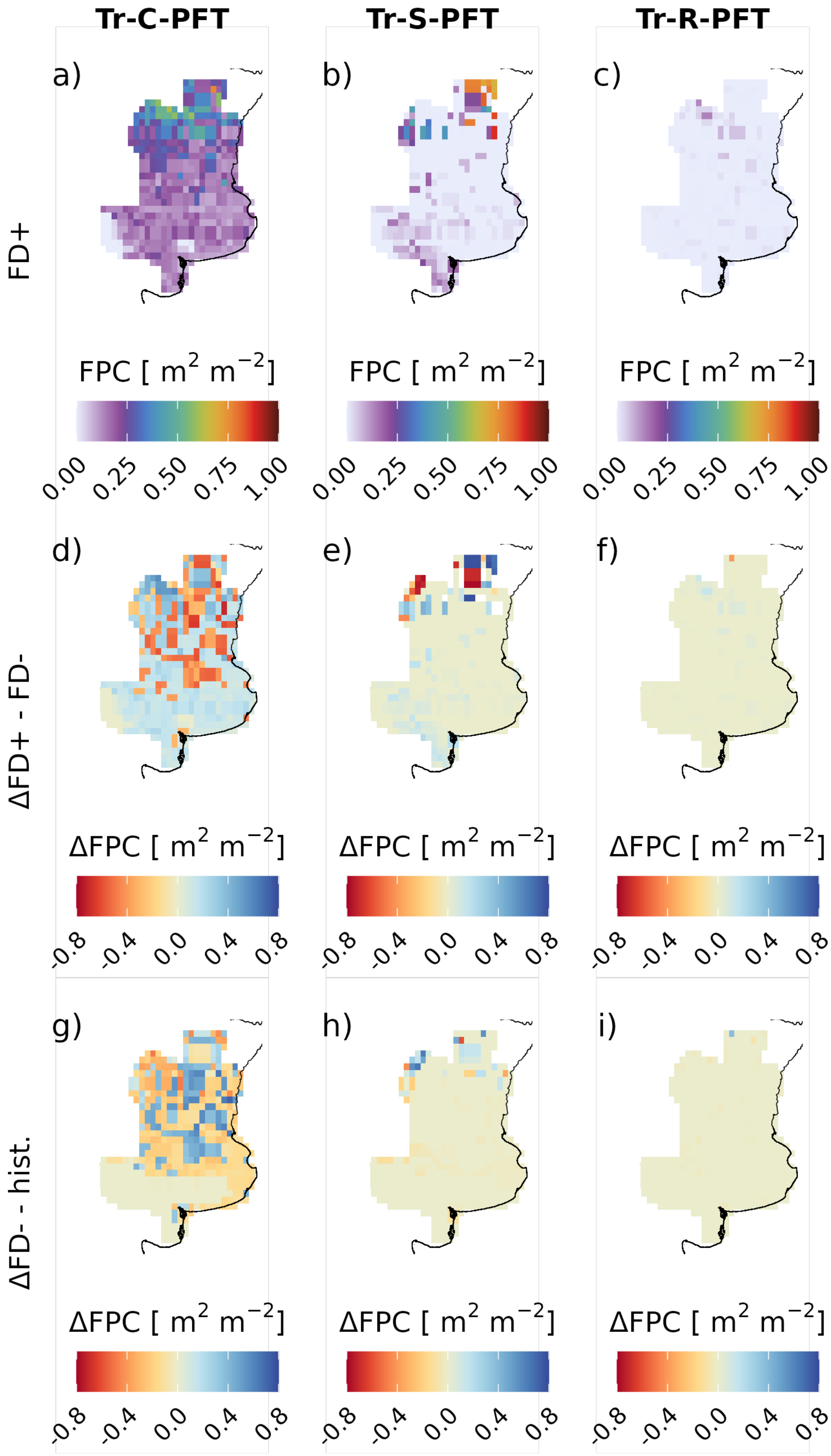

Fig. S19. Distribution of the absolute foliage projective cover (a,b,c) and relative difference between FD+ and FD- (c,d,e) and end of the historical time period (g,h,i) for the tropical C- (a,d,g), S- (b,e,h) and R-PFT (c,f,i) for the Argentinian Pampas. All distribution are based on the 2005 to 2014 and 2091 to 2100 average of the respective output variable simulated using climate data from the MRI-ESM2-0.

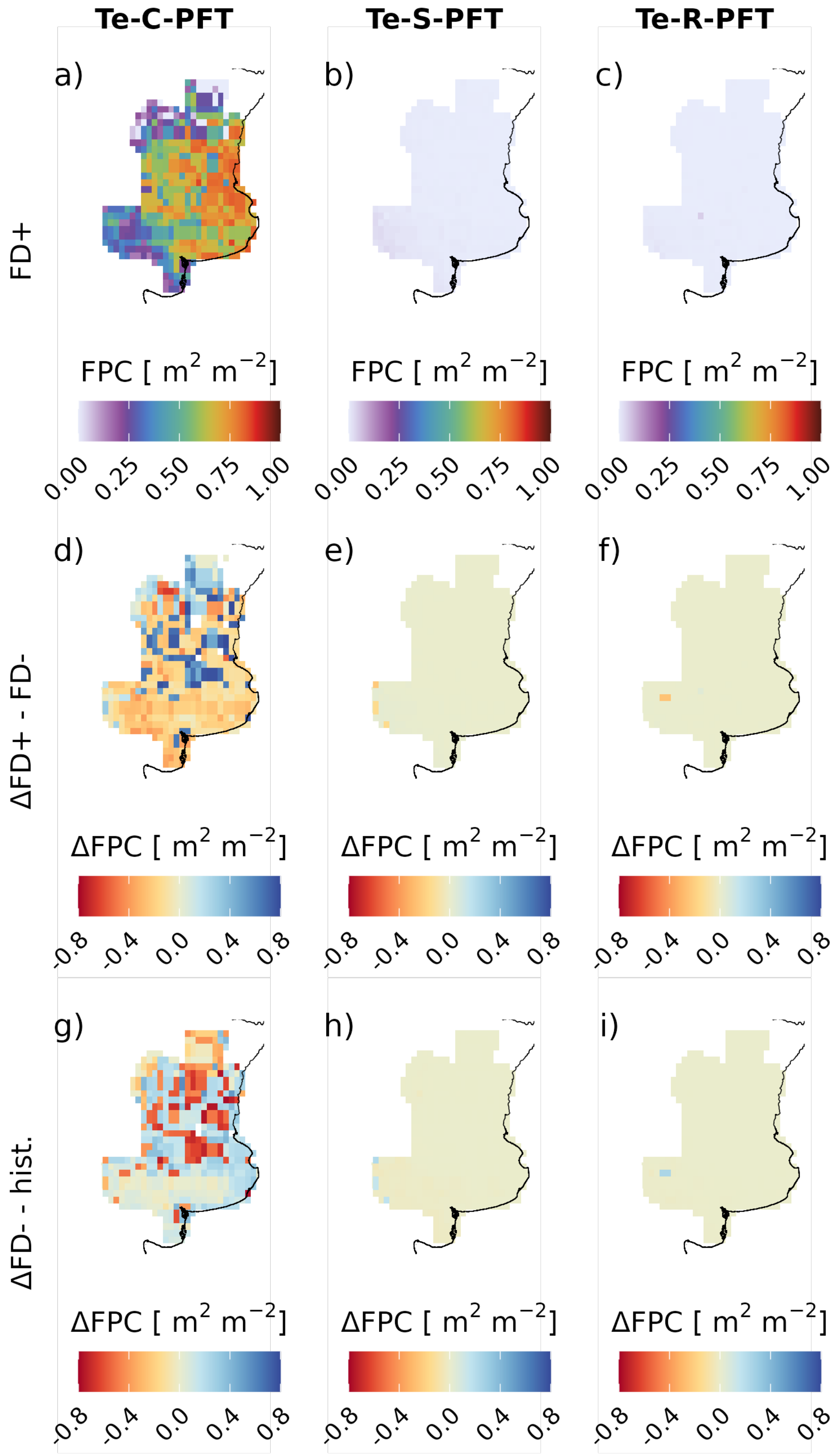

Fig. S20. Distribution of the absolute foliage projective cover (a,b,c) and relative difference between FD+ and FD- (c,d,e) and end of the historical time period (g,h,i) for the temperate C- (a,d,g), S- (b,e,h) and R-PFT (c,f,i) for the Argentinian Pampas. All distribution are based on the 2005 to 2014 and 2091 to 2100 average of the respective output variable simulated using climate data from the MRI-ESM2-0.

References

1. GD Farquhar, S von Caemmerer, JA Berry, A biochemical model of photosynthetic CO2 assimilation in leaves of C3 species. *Planta* **149**, 78–90 (1980).
2. G Collatz, M Ribas-Carbo, J Berry, Coupled Photosynthesis-Stomatal Conductance Model for Leaves of C 4 Plants. *Funct. Plant Biol.* **19** (1992).
3. GJ Collatz, JT Ball, C Grivet, JA Berry, Physiological and environmental regulation of stomatal conductance, photosynthesis and transpiration: A model that includes a laminar boundary layer. *Agric. For. Meteorol.* **54**, 107–136 (1991).
4. A Haxeltine, IC Prentice, A General Model for the Light-Use Efficiency of Primary Production. *Funct. Ecol.* **10**, 551–561 (1996).
5. S Sitch, et al., Evaluation of ecosystem dynamics, plant geography and terrestrial carbon cycling in the LPJ dynamic global vegetation model. *Glob. Chang. Biol.* **9**, 161–185 (2003).
6. A Haxeltine, IC Prentice, BIOME3: An equilibrium terrestrial biosphere model based on ecophysiological constraints, resource availability, and competition among plant functional types. *Glob. Biogeochem. Cycles* **10**, 693–709 (1996).
7. M Forkel, et al., Identifying environmental controls on vegetation greenness phenology through model–data integration. *Biogeosciences* **11**, 7025–7050 (2014).
8. SB Wirth, et al., Connecting competitor, stress-tolerator and ruderal (CSR) theory and Lund Potsdam Jena managed Land 5 (LPJmL 5) to assess the role of environmental conditions, management and functional diversity for grassland ecosystem functions. *Biogeosciences* **21**, 381–410 (2024).
9. W von Bloh, et al., Implementing the nitrogen cycle into the dynamic global vegetation, hydrology, and crop growth model LPJmL (version 5.0). *Geosci. Model. Dev.* **11**, 2789–2812 (2018).
10. SB Wirth, et al., Biological nitrogen fixation of natural and agricultural vegetation simulated with LPJmL 5.7.9. *EGUsphere* pp. 1–29 (2024).
11. J Heinke, S Rolinski, C Müller, Modelling the role of livestock grazing in C and N cycling in grasslands with LPJmL5.0-grazing. *Geosci. Model. Dev.* **16**, 2455–2475 (2023).
12. IJ Wright, et al., The worldwide leaf economics spectrum. *Nature* **428**, 821–827 (2004).
13. RB Jackson, et al., A global analysis of root distributions for terrestrial biomes. *Oecologia* **108**, 389–411 (1996).
14. J Heinke, A livestock density input for LPJmL (2025).
15. M Herrero, et al., Biomass use, production, feed efficiencies, and greenhouse gas emissions from global livestock systems. *Proc. Natl. Acad. Sci.* **110**, 20888–20893 (2013).
16. DD Briske, Plant response to defoliation: Morphological considerations and allocation priorities in *Rangelands: A Resource under Siege*, eds. Joss, P. J., Lynch P. W., Williams O. B. (Aust. Acad. Sci., Canberra), pp. 425–427 (1986).
17. G Stuart-Hill, M Mentis, Coevolution of African grasses and large herbivores. *Proc. Annu. Congr. Grassl. Soc. South. Afr.* **17**, 122–128 (1982).
18. Y Zhang, et al., A global moderate resolution dataset of gross primary production of vegetation for 2000–2016. *Sci Data* **4**, 170165 (2017).
19. S Wolf, et al., Citizen science plant observations encode global trait patterns. *Nat Ecol Evol* **6**, 1850–1859 (2022).
20. J Lee, et al., Future Global Climate: Scenario-Based Projections and Near-Term Information in *Climate Change 2021: The Physical Science Basis. Contribution of Working Group I to the Sixth Assessment Report of the Intergovernmental Panel on Climate Change*, eds. V Masson-Delmotte, et al. (Cambridge University Press, Cambridge, United Kingdom and New York, NY, USA), pp. 553–672 (2021).
